# Supplementary material for: Replicated anthropogenic hybridisations reveal parallel patterns of admixture in marine mussels
Source: Evol Appl. 2019 Nov 22;13(3):575–99. doi: 10.1111/eva.12879 (PMC7045717; doi:10.1111/eva.12879)
Supplement: Supplementary file 1 [file EVA-13-575-s001.pdf]

Replicated anthropogenic hybridisations reveal parallel patterns of admixture in marine mussels.

September 27, 2019

Supplementary information

Contents

1 Admixture time **3**

2 Supplementary Figures **4**

3 Supplementary Tables **30**

List of Figures

S1 Complete sampling map 4
S2 Kingfisher extraction program 5
S3 HWE 6
S4 `Admixture` for reference populations 6
S5 Complete `Structure` 7
S6 `Structure` $K = 4$ 8
S7 `Structure` lab crosses 8
S8 `Structure` Le Havre 9
S9 `Structure` Cherbourg 9
S10 `Structure` Saint-Malo 9
S11 `Structure` bay of Brest 10
S12 `Structure` Saint-Nazaire 10
S13 `Structure` human structures North-Sea 10
S14 `Structure` naturally admixed and Norway admixed 11
S15 `Structure` other populations 11
S16 Map of La Rochelle 12
S17 Map of Bilbao 12
S18 Map of the Long Island Sound 13
S19 `Structure` without admixture model 14
S20 `Structure` without admixture model, ports and local populations 15
S21 `Structure` of selected individuals in each admixed category 16
S22 Allele frequencies 17
S23 Ancestry estimates distributions 17
S24 LD 18

S25 Newhybrids 1 19
S26 Newhybrids 2 20
S27 Newhybrids 3 21
S28 Allele frequency clines Le Havre-North 22
S29 Allele frequency clines Le Havre-South 22
S30 Allele frequency clines Cherbourg 23
S31 Allele frequency clines Saint-Nazaire 23
S32 Allele frequency clines Brest 24
S33 Summary of the correlations of distortions between populations 25
S34 Pairwise scatter plots of distorsion comparisons 26
S35 Correlation matrix for temporal variation in Cherbourg. 27
S36 Summary of the correlations of distortions between populations in Cherbourg 27
S37 Pairwise scatter plots of distorsion comparisons in Cherbourg 28
S38 Frequencies of the mitochondrial marker 29
S39 Newspaper article about the Swansea King's dock mussels 29

List of Tables

S1 Population information 30
S2 Softwares and R packages used in this study 37
S3 Analyses thresholds 38
S4 Reference panel groups and populations 39
S5 Genetic map 40
S6 Fst values between reference groups. 41
S7 Fst values and significance between ports. 41
S8 Fst values and significance between Cherbourg years. 41
S9 Fst values and significance between naturally and Norway admixed. 41
S10 Statistical tests of ancestry comparisons in naturally and Norway admixed 42
S11 Statistical tests of ancestry comparisons in dock mussels 43
S12 Statistical tests of ancestry comparisons in Cherbourg 43
S13 Statistical tests of ancestry comparisons in Brittany 44
S14 Admixture time estimation 45

1 Admixture time

To estimate simply admixture time, `Structure` runs were used as indicated in Falush, Stephens, and Pritchard (2003) using the linkage model. For each port (or year for Cherbourg) only admixed dock mussels individuals were considered with additional reference populations being edu_eu_south and gallo_med. The popflag column was set to 1 for reference individuals to better estimate the parental allele frequencies. Only markers on the genetic map were used to avoid bias through undetected linkage with markers that could not be included in the genetic map analysis. Options used: LINKAGE = 1, PFROMPOPFLAGONLY = 1, NOADMIX = 0, LOG10RSTART = -2, LOG10RMIN = -3, LOG10RMAX = 2, LOG10RPROPSD = 0.1. See the parameter files for more details. Results of average r for 25 runs are presented in table S14, the number of generations is given by $r \times 100$ as genetic distance are given in cM.

2 Supplementary Figures

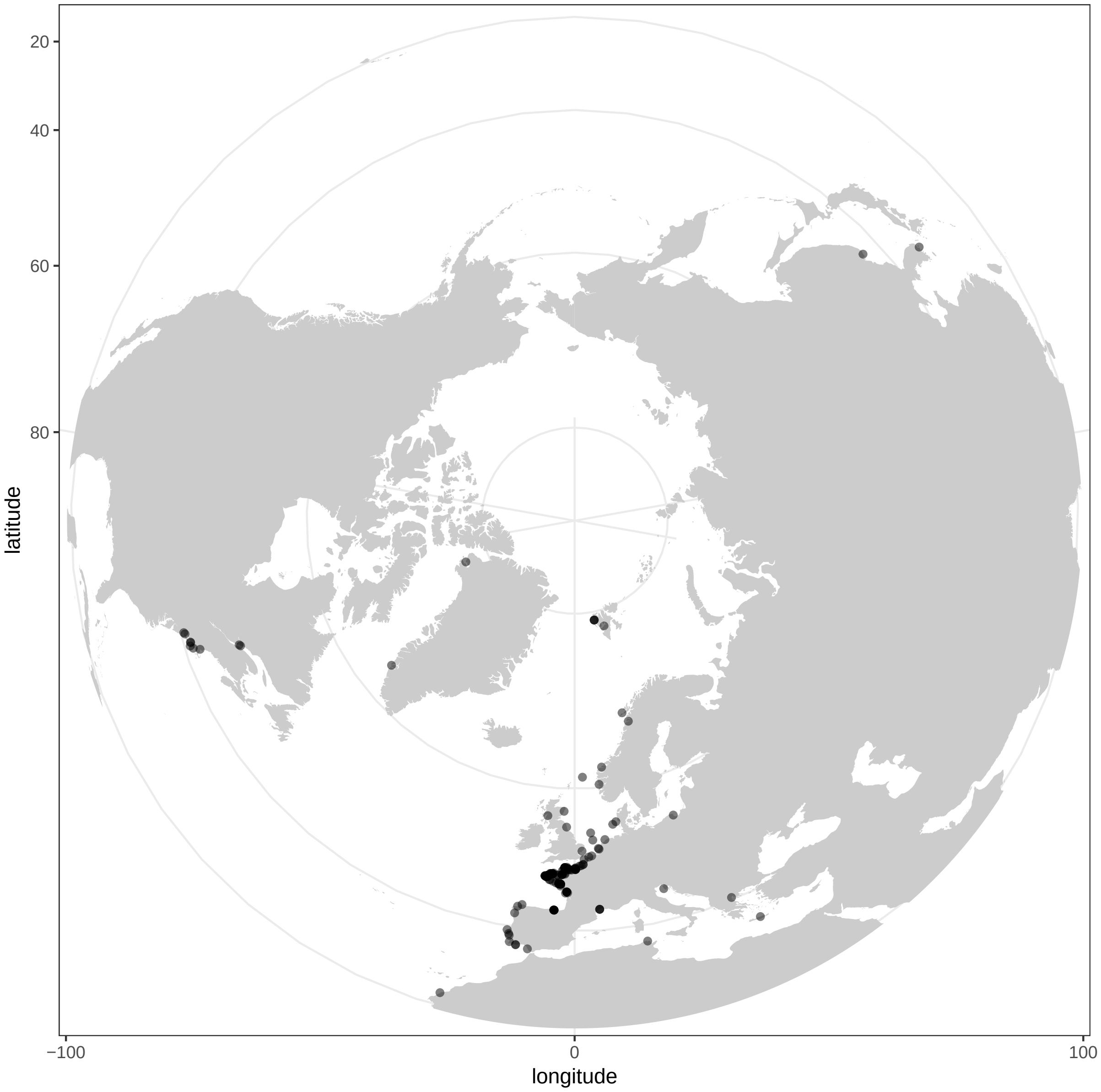

Figure S1: Location of all samples considered in the study.

Steps data

Tip1		96 DW tip comb	
Pick-Up		Tip Plate	
Bind		Sample	
	Beginning of step	Precollect	No
		Release time, speed	00:00:30, Bottom mix
	Mixing / heating:	Mixing time, speed	00:04:30, Half mix
		Heating during mixing	No
	End of step	Postmix	No
		Collect count	3
		Collect time [s]	1
Wash 1		MB3	
	Beginning of step	Precollect	No
		Release time, speed	00:00:10, Bottom mix
	Mixing / heating:	Mixing time, speed	00:01:00, Half mix
		Heating during mixing	No
	End of step	Postmix	No
		Collect count	3
		Collect time [s]	1
Wash 2		MB4	
	Beginning of step	Precollect	No
		Release time, speed	00:00:10, Bottom mix
	Mixing / heating:	Mixing time, speed	00:01:00, Half mix
		Heating during mixing	No
	End of step	Postmix	No
		Collect count	3
		Collect time [s]	1
Wash 3		MB5	
	Beginning of step	Precollect	No
		Release beads	No
	Mixing / heating:	Mixing time, speed	00:00:30, Slow
		Heating during mixing	No
	End of step	Postmix	No
		Collect beads	No
Elution		Elution	
	Beginning of step	Precollect	No
		Release time, speed	00:00:15, Bottom mix
	Mixing / heating:	Shake 1 time, speed	00:01:00, Medium
		Shake 2 time, speed	00:00:10, Bottom mix
		Loop count	8
		Heating temperature [°C]	72
		Preheat	Yes
	End of step	Postmix	No
		Collect count	3
		Collect time [s]	30
ReleaseBeads1		MB5	
		Release time, speed	00:00:10, Half mix
Leave		MB5	

Figure S2: Program for DNA extraction with the Kingfisher Flex robot.

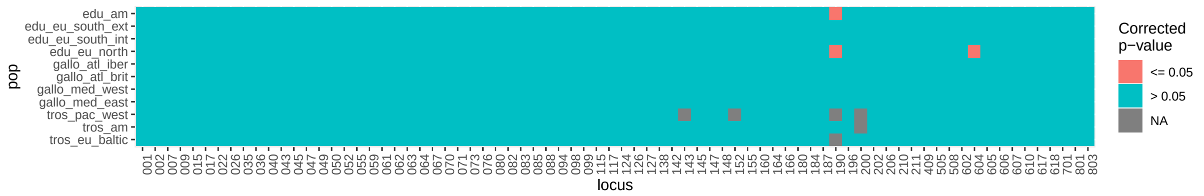

Figure S3: Hardy-Weinberg equilibrium test in each reference population. Benjamini-Yekutieli corrected p values are used.

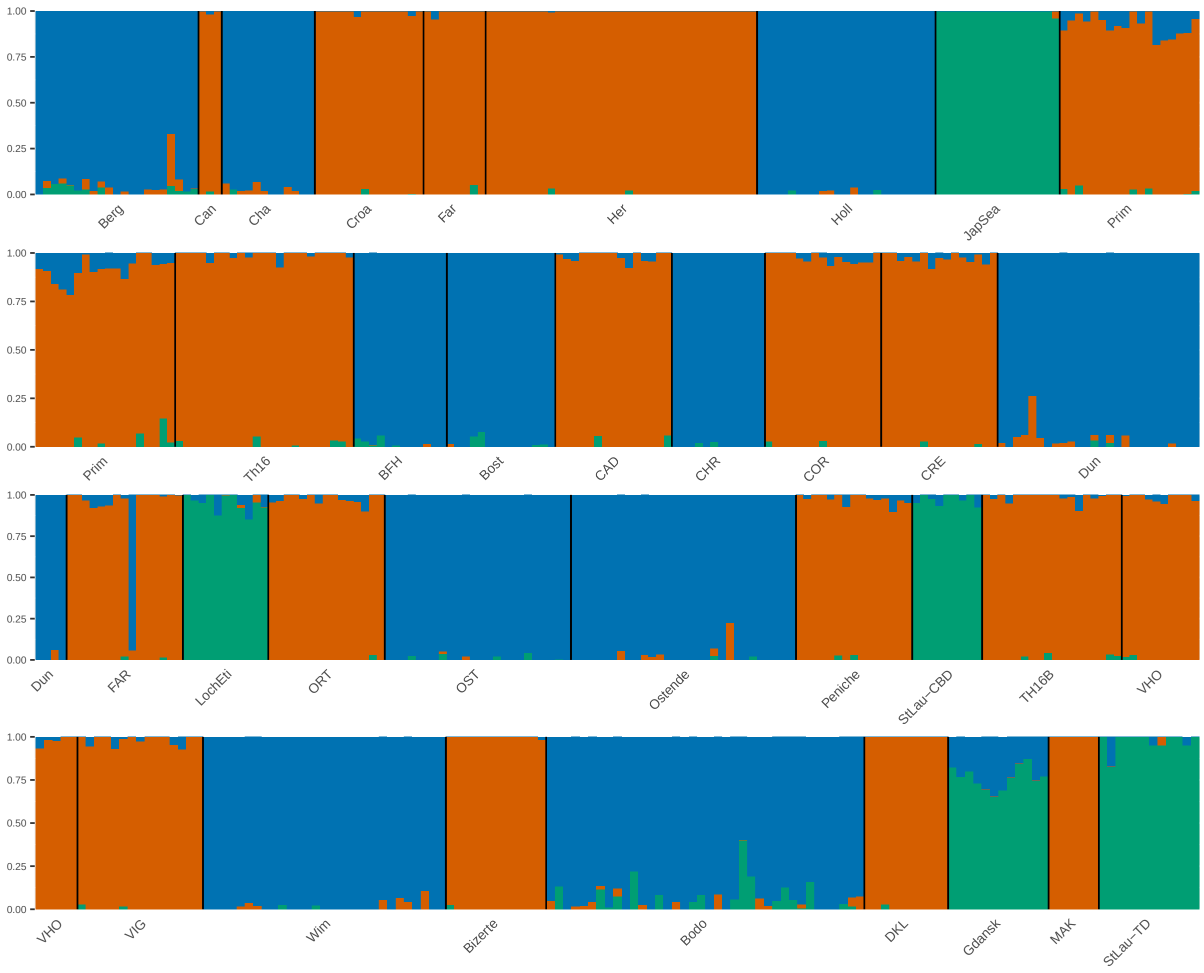

Figure S4: `Admixture` results for reference groups, $K = 3$, before filtration.

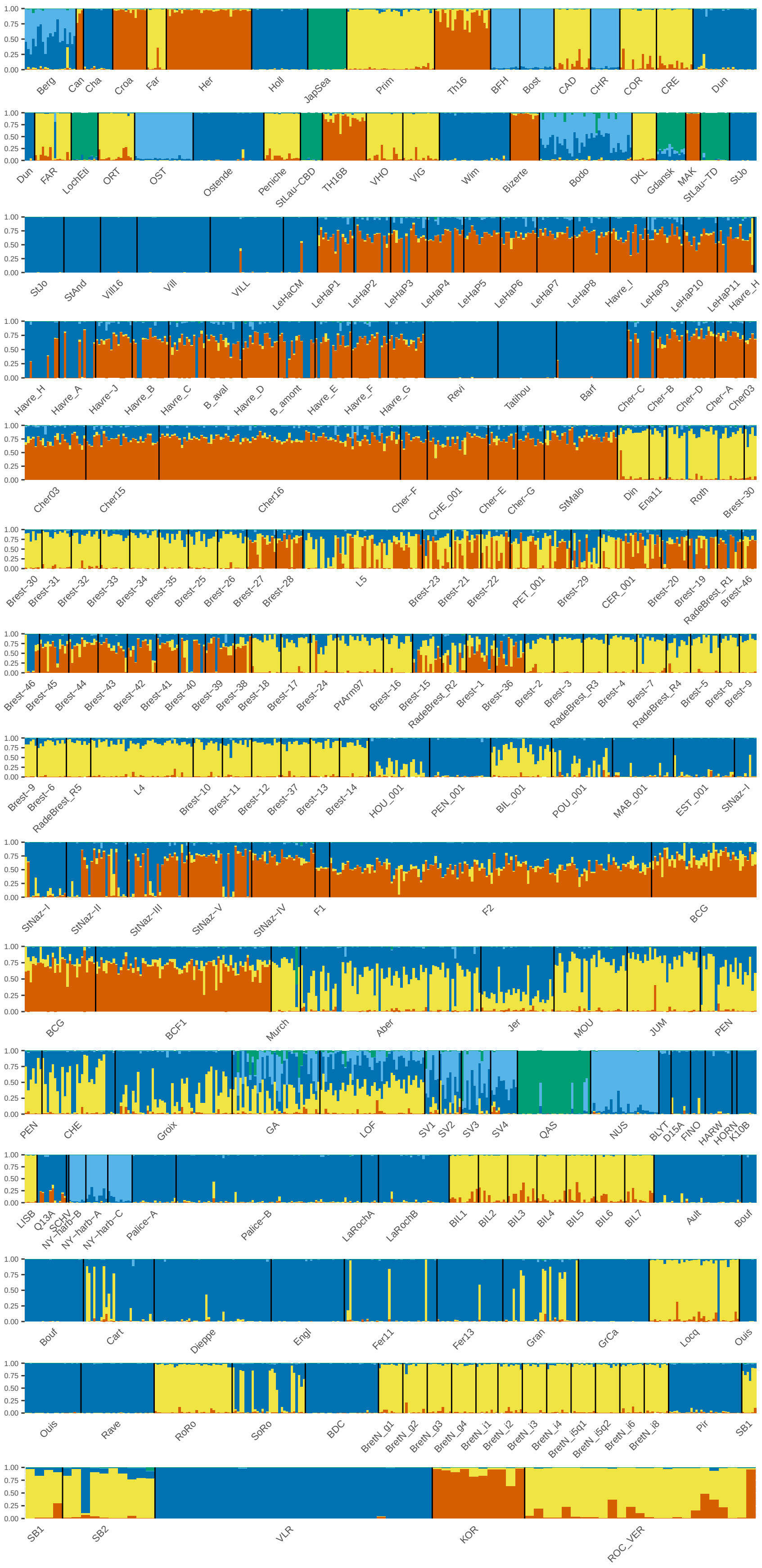

Figure S5: `Structure` results with all individuals, $K = 6$ major cluster.

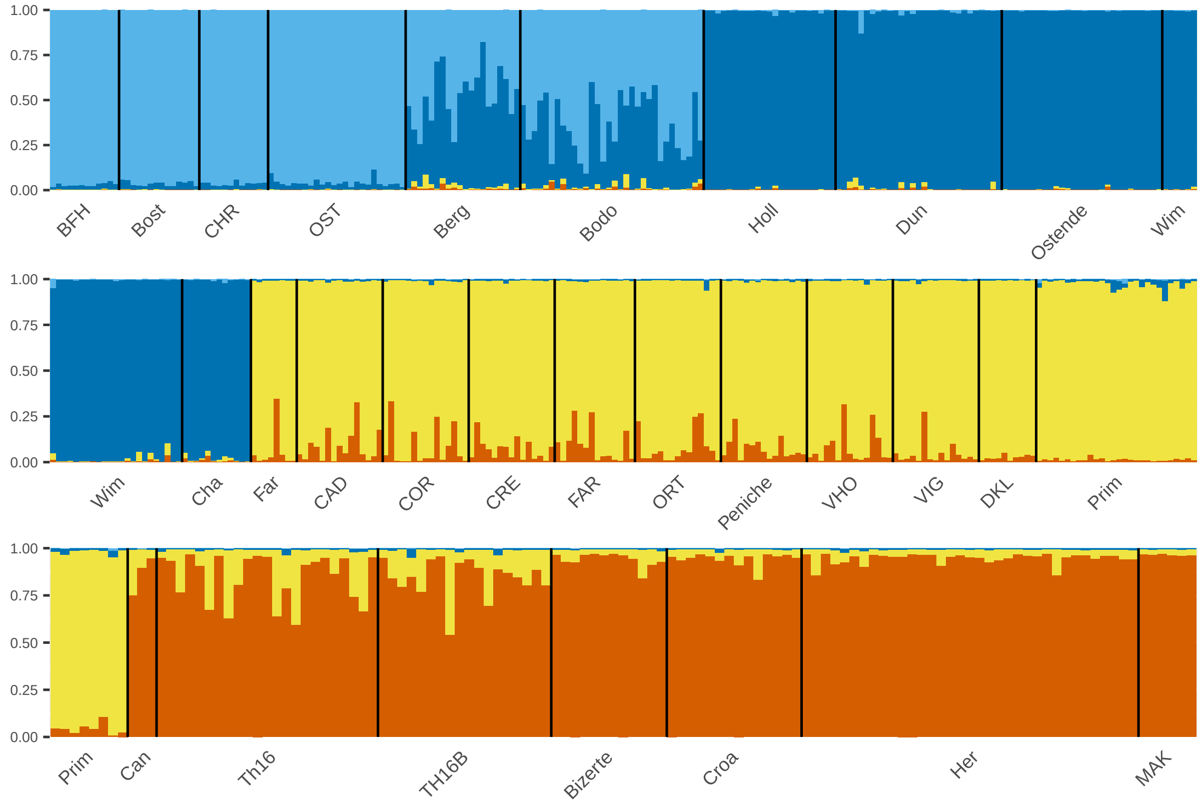

Figure S6: `Structure` results with all individuals excluding the ones with *M. trossulus* ancestry, $K = 4$ major cluster. Subset corresponding to reference populations only including individuals from the reference groups (table S4) after filtration.

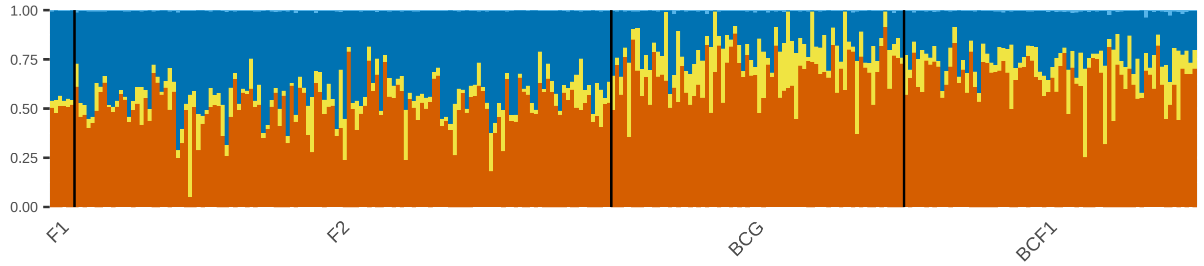

Figure S7: Subset of the lab crosses.

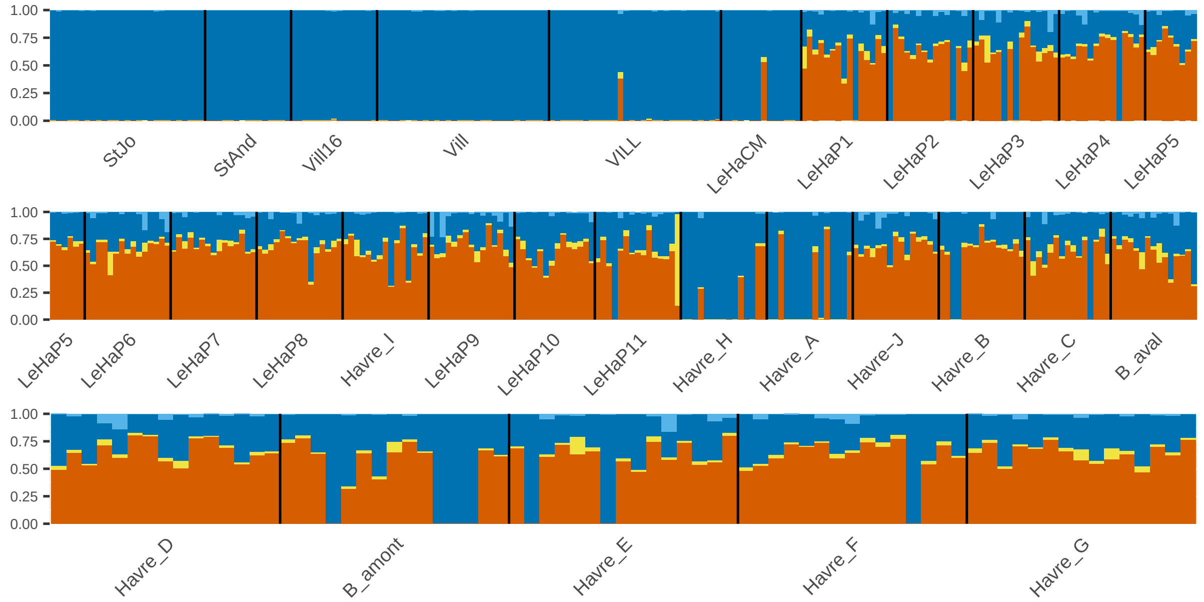

Figure S8: Subset of the port of Le Havre.

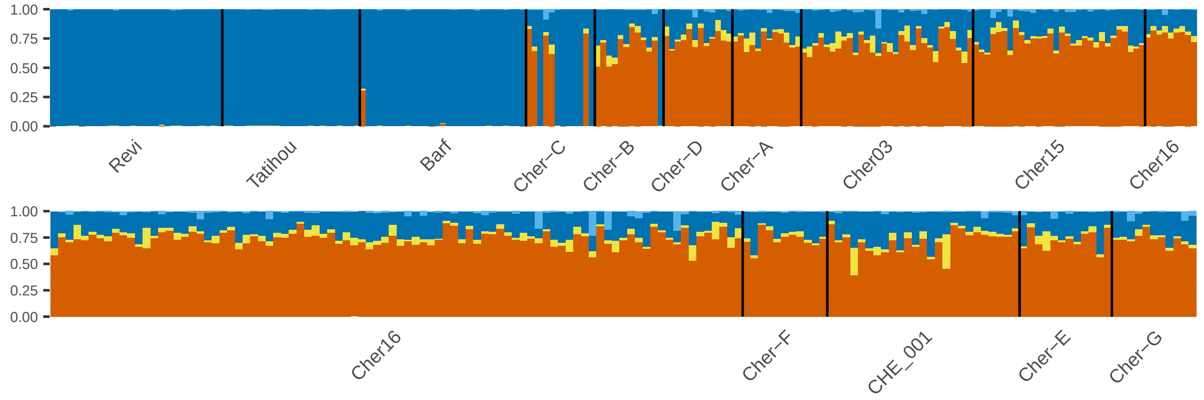

Figure S9: Subset of the port of Cherbourg.

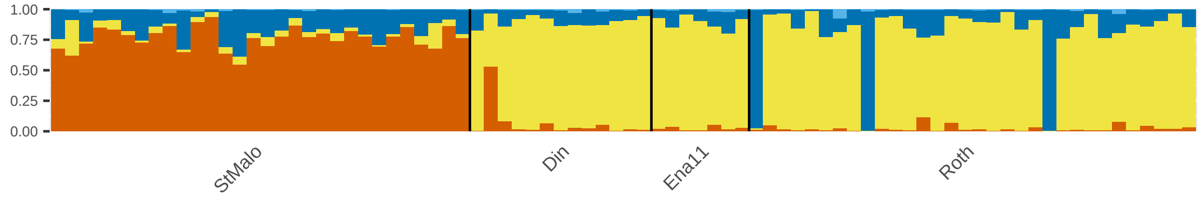

Figure S10: Subset of the port of Saint-Malo.

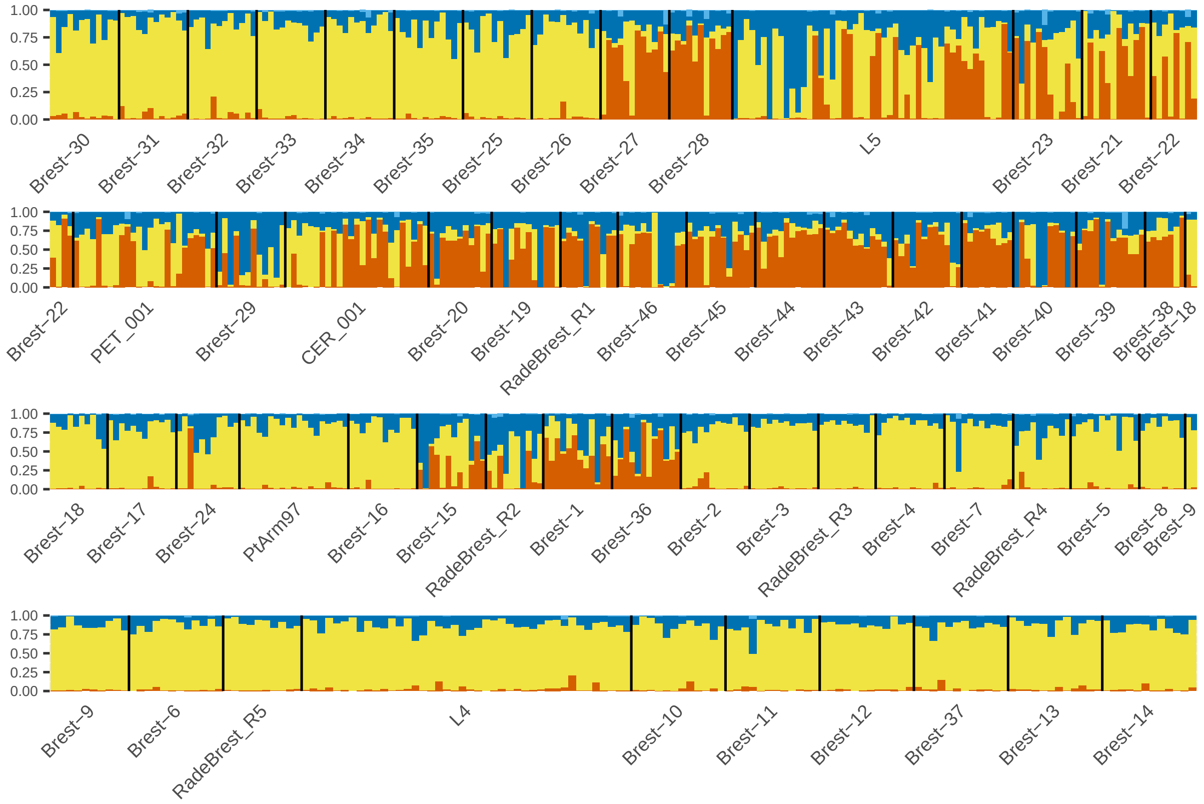

Figure S11: Subset of the bay of Brest area.

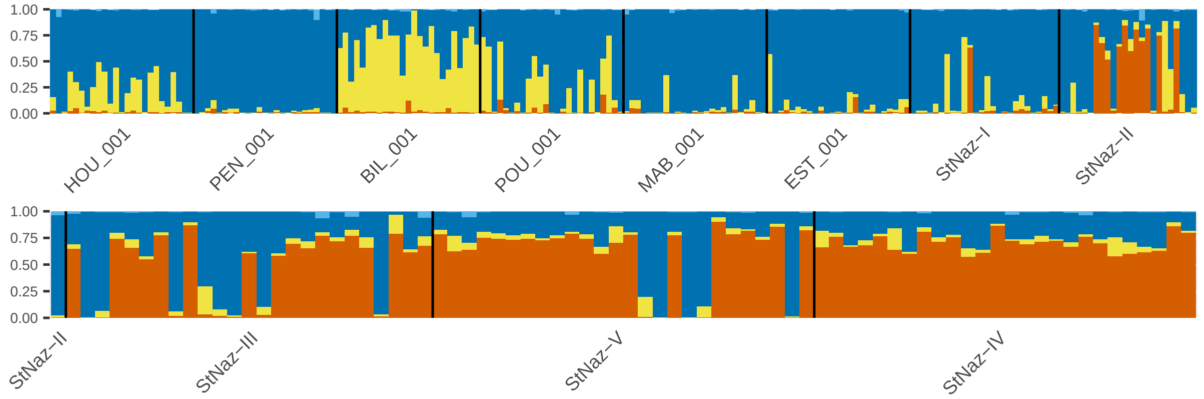

Figure S12: Subset of the port of Saint-Nazaire.

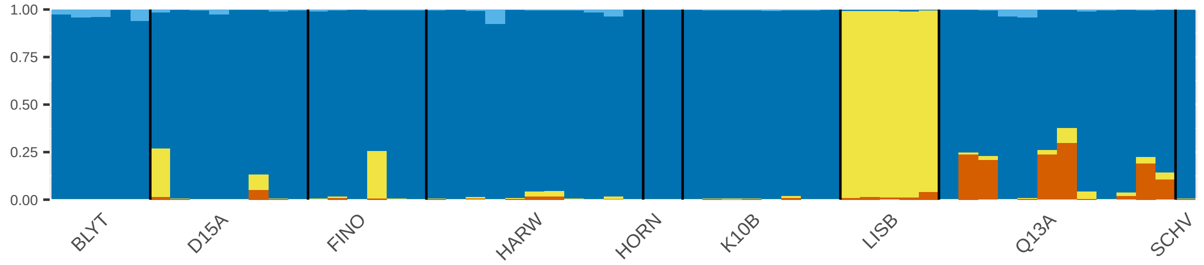

Figure S13: Subset of the populations studied in Coolen (2017).

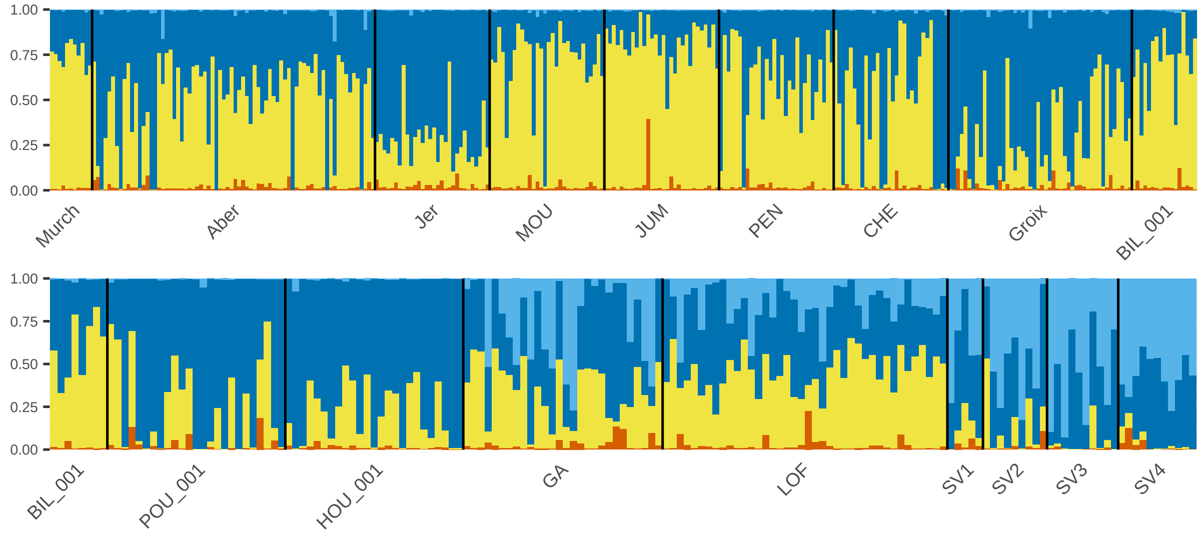

Figure S14: Subset of admixed populations with Atlantic *M. galloprovincialis* ancestries, including Natural admixed and Norway admixed. Populations LOF and SV1 to SV4 come from the Mathiesen et al. (2016) study.

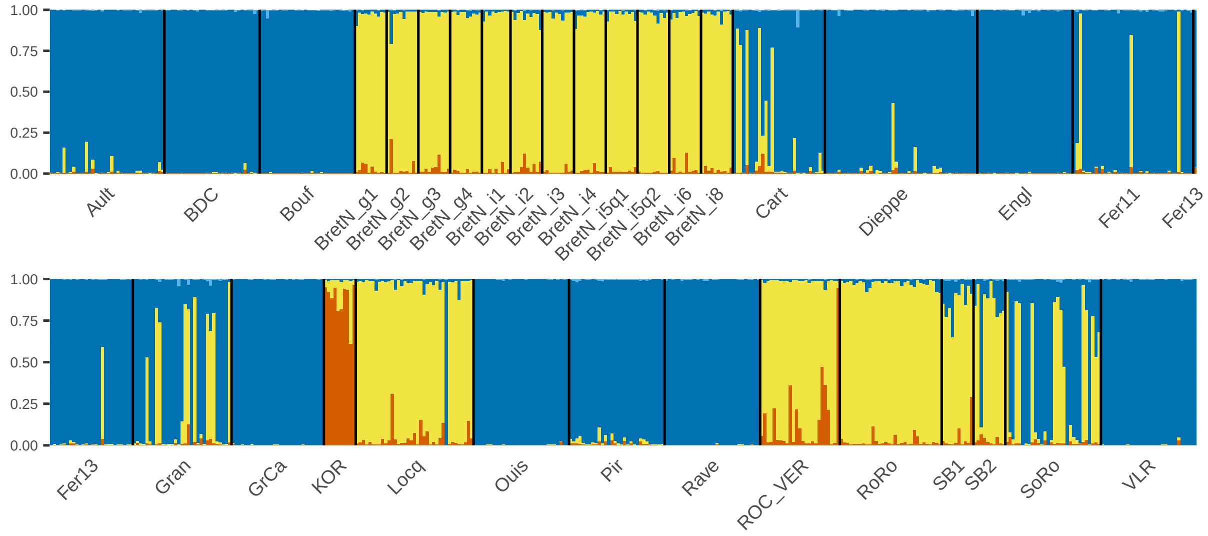

Figure S15: Subset of additional populations.

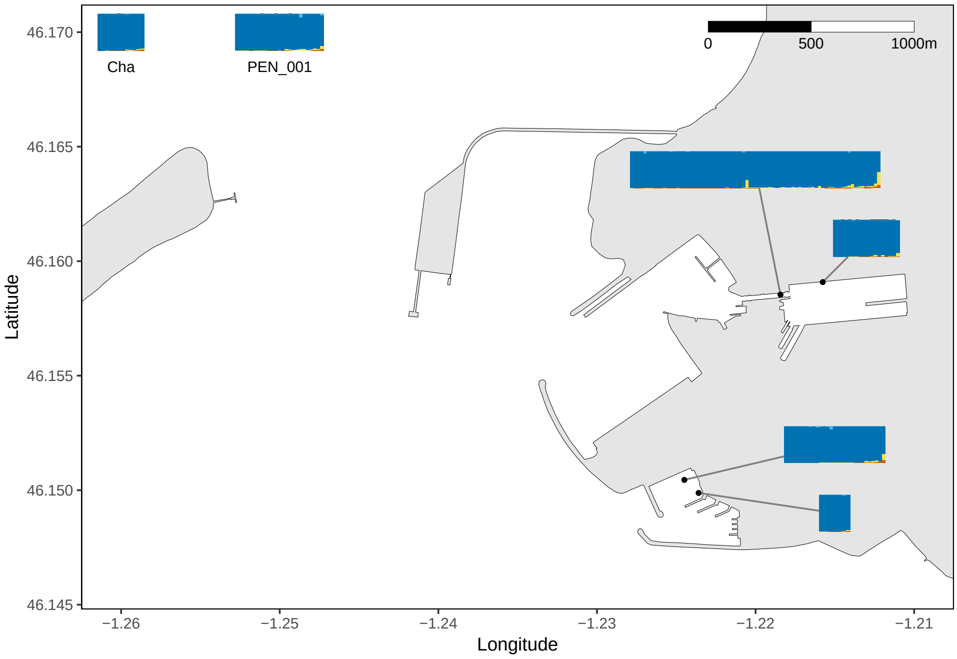

Figure S16: Map of the port of La Rochelle, bay of Biscay, France. Barplots represent individual ancestry proportions for each site. Barplots at the map edges correspond to distant populations. No Mediterranean *M. galloprovincialis* ancestry is detected in this port.

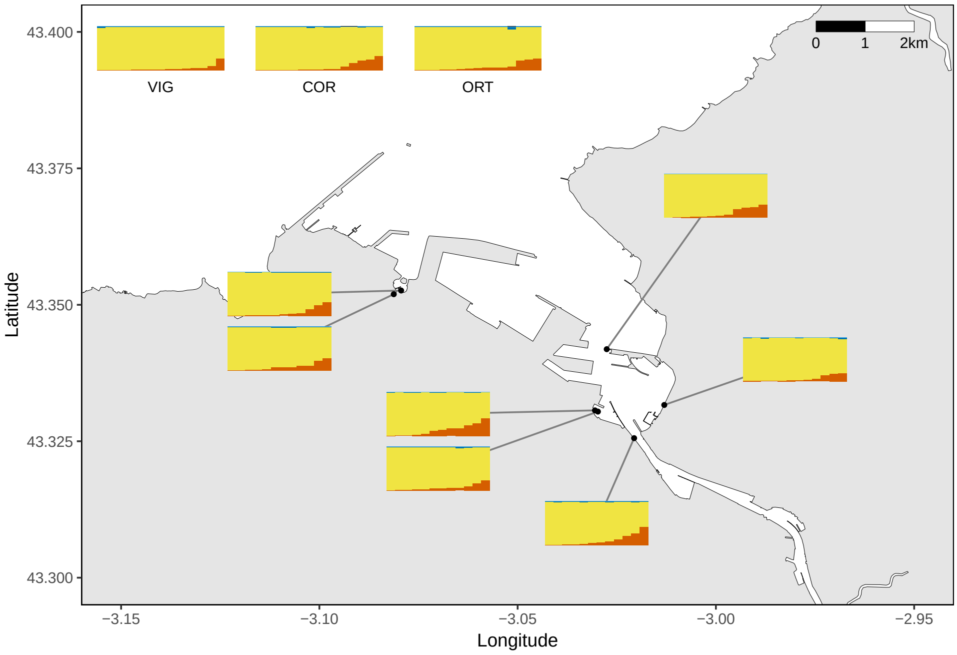

Figure S17: Map of the port of Bilbao, Basque Country, Spain. Sites inside the port do not exhibit different ancestriy compositions than more distant populations

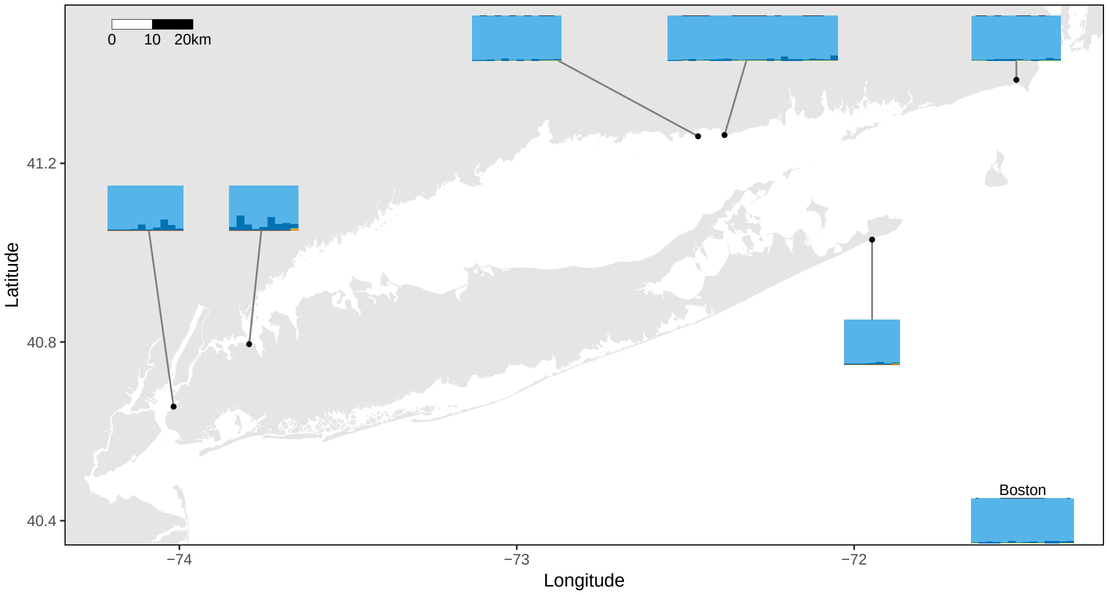

Figure S18: Map of the Long Island Sound, NY, USA. The two leftmost populations exhibit more European *M. edulis* ancestry than local American *M. edulis* populations.

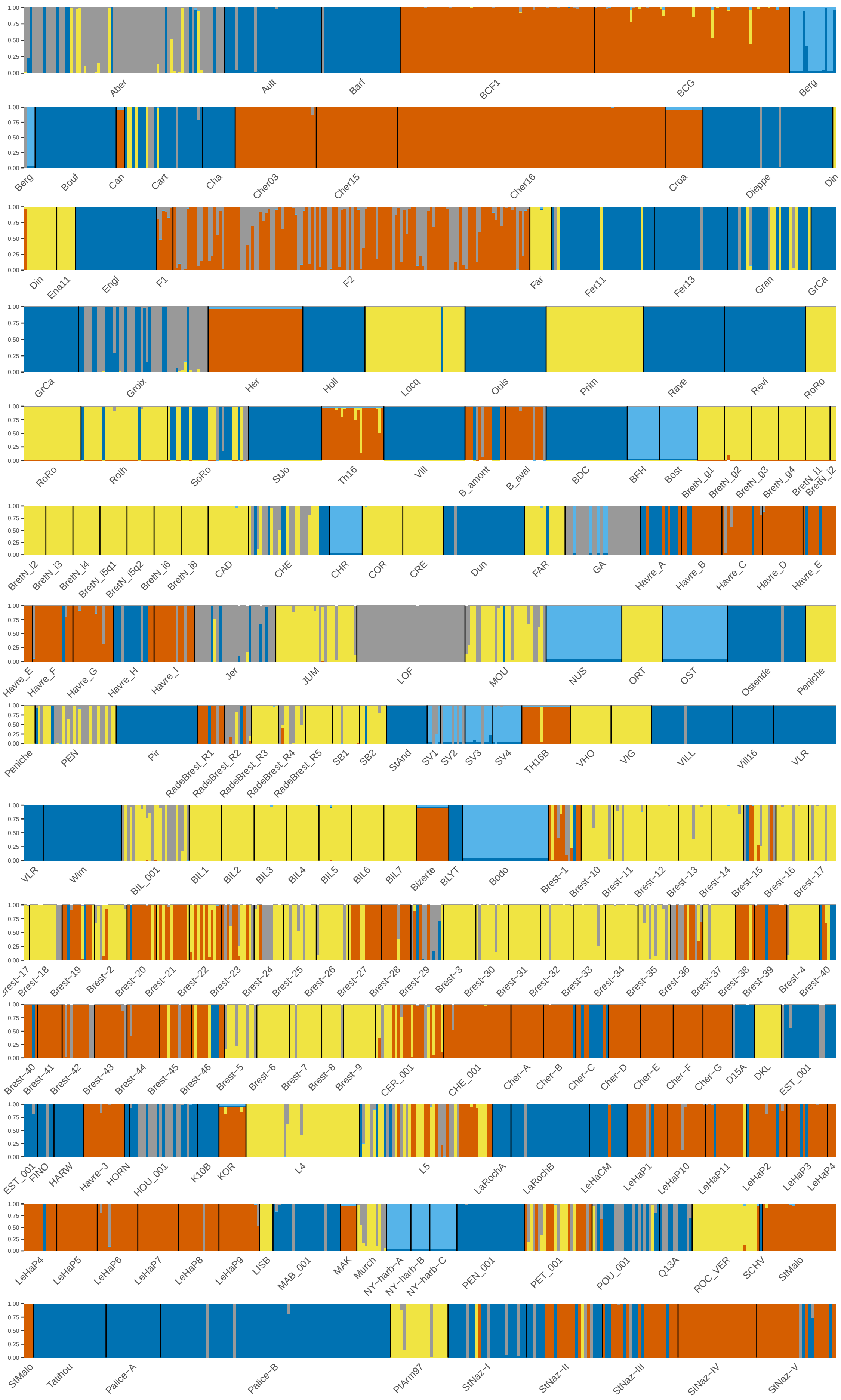

Figure S19: `Structure` without admixture model, $K = 5$

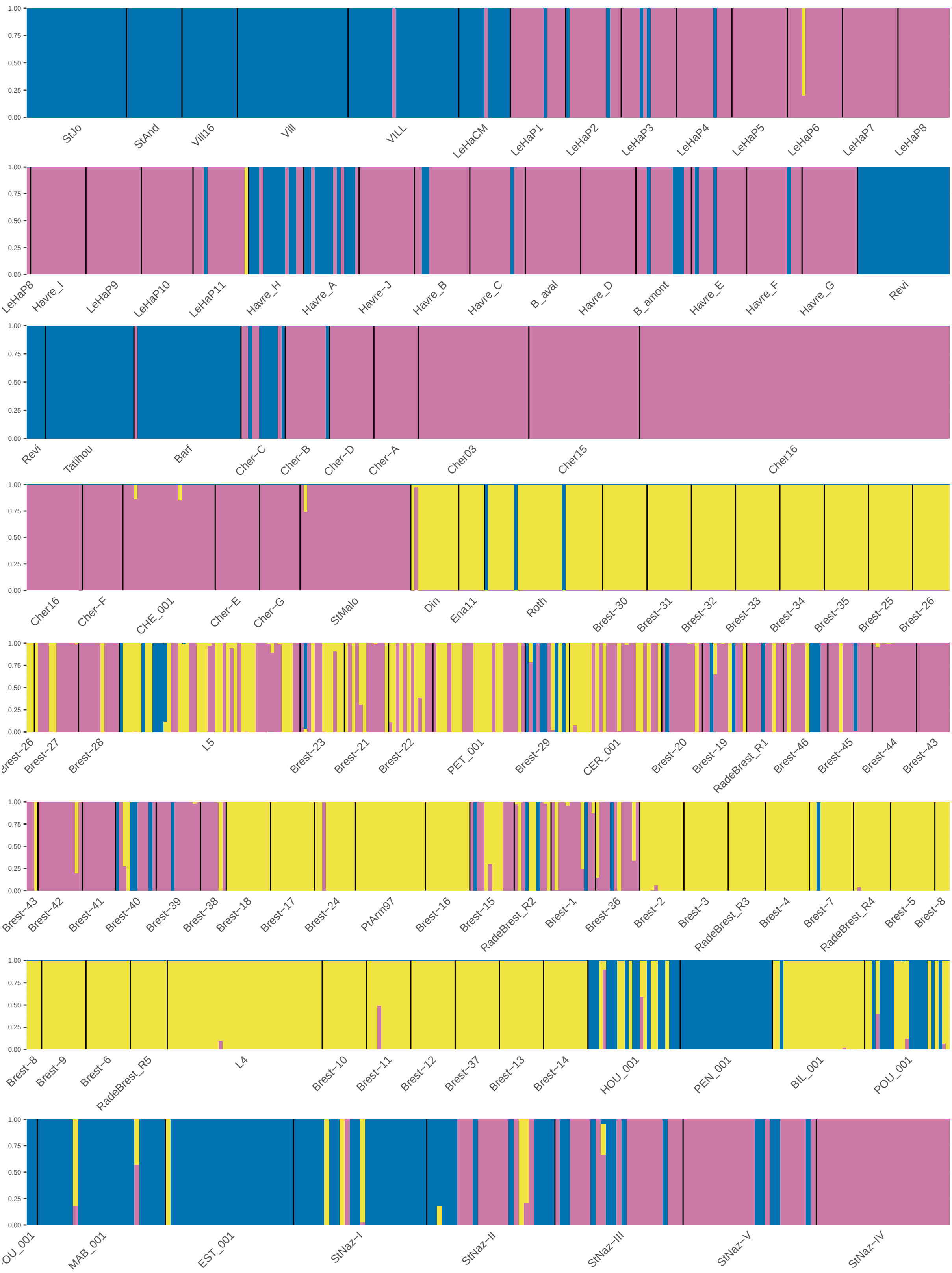

Figure S20: `Structure` without admixture model only with port and local populations, $K = 3$. This output is used to classify dock mussels for downstream analyses.

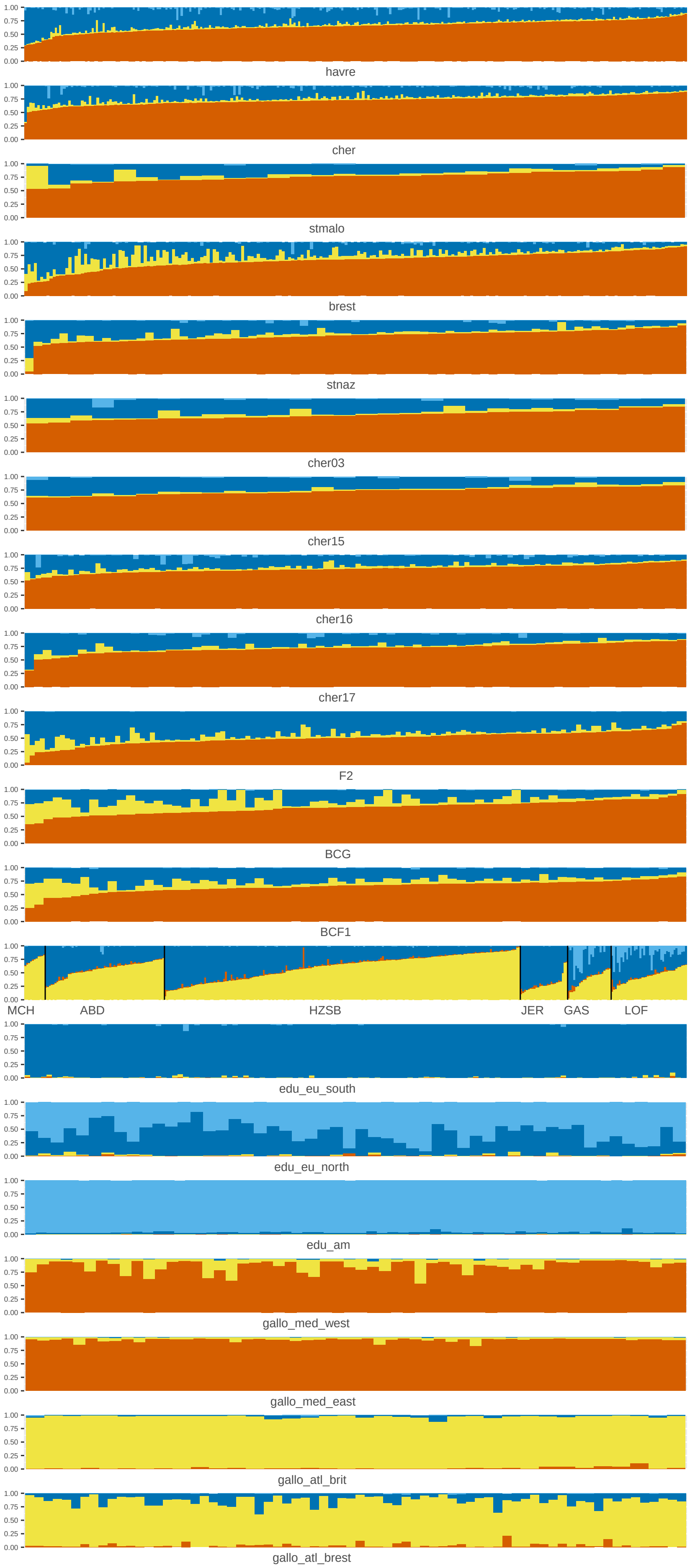

Figure S21: Structure plot of selected individuals for each group for downstream analyses

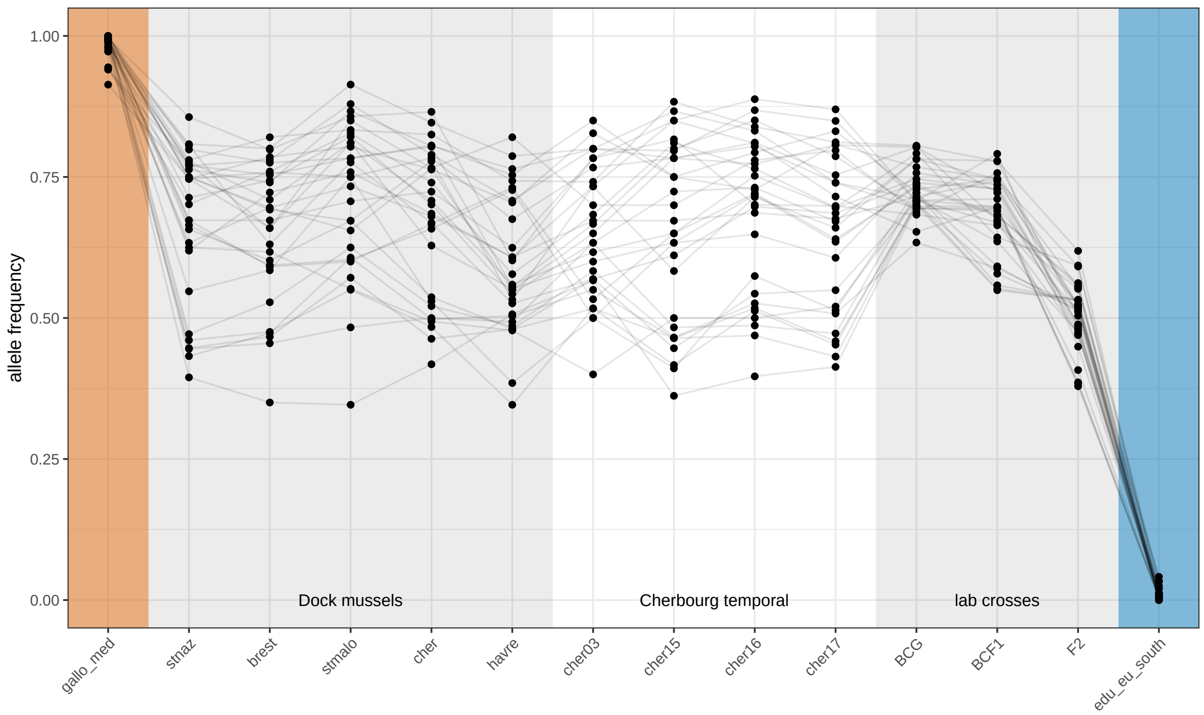

Figure S22: Allele frequencies of differentiated markers (AFD > 0.9) between Med. *M. galloprovincialis* and South-Eu. *M. edulis* in dock mussels, temporal samples of Cherbourg and laboratory crosses. Markers not heterozygous in the F1 cross were removed.

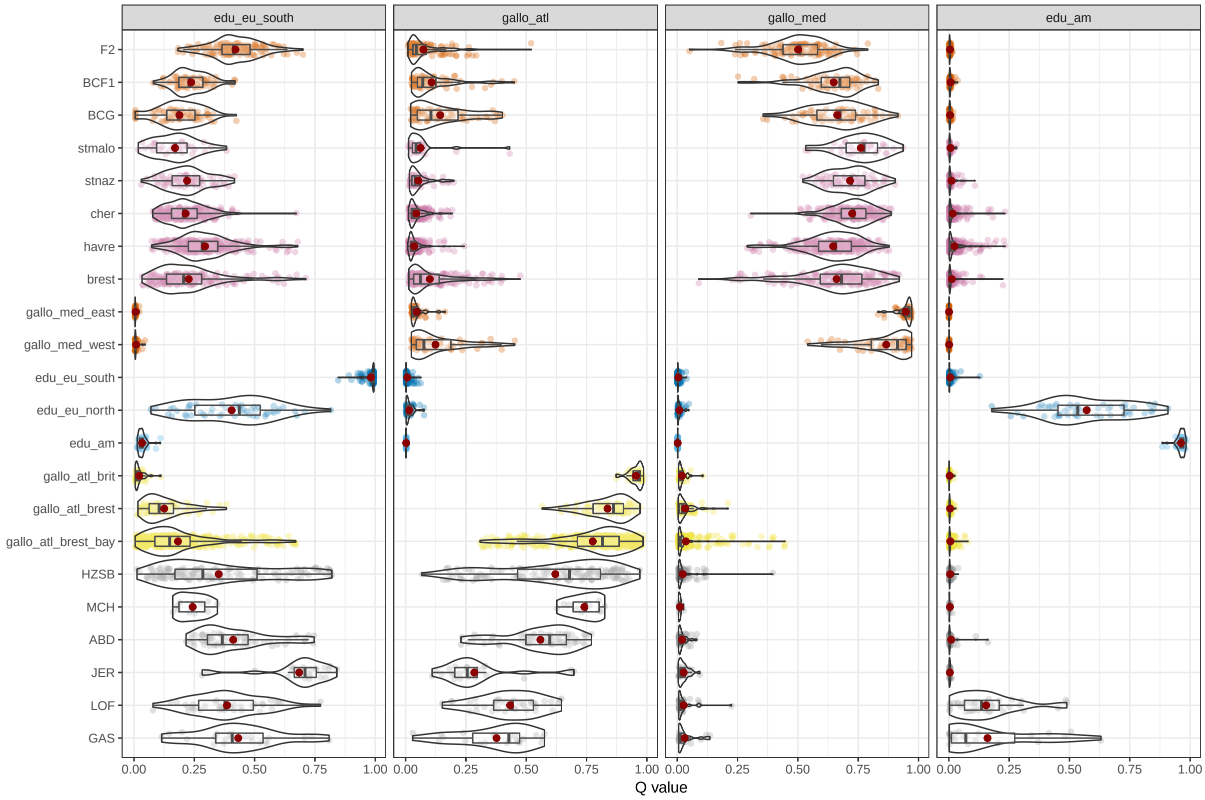

Figure S23: Ancestry levels for each `Structure` cluster in populations of admixed mussels and reference groups.

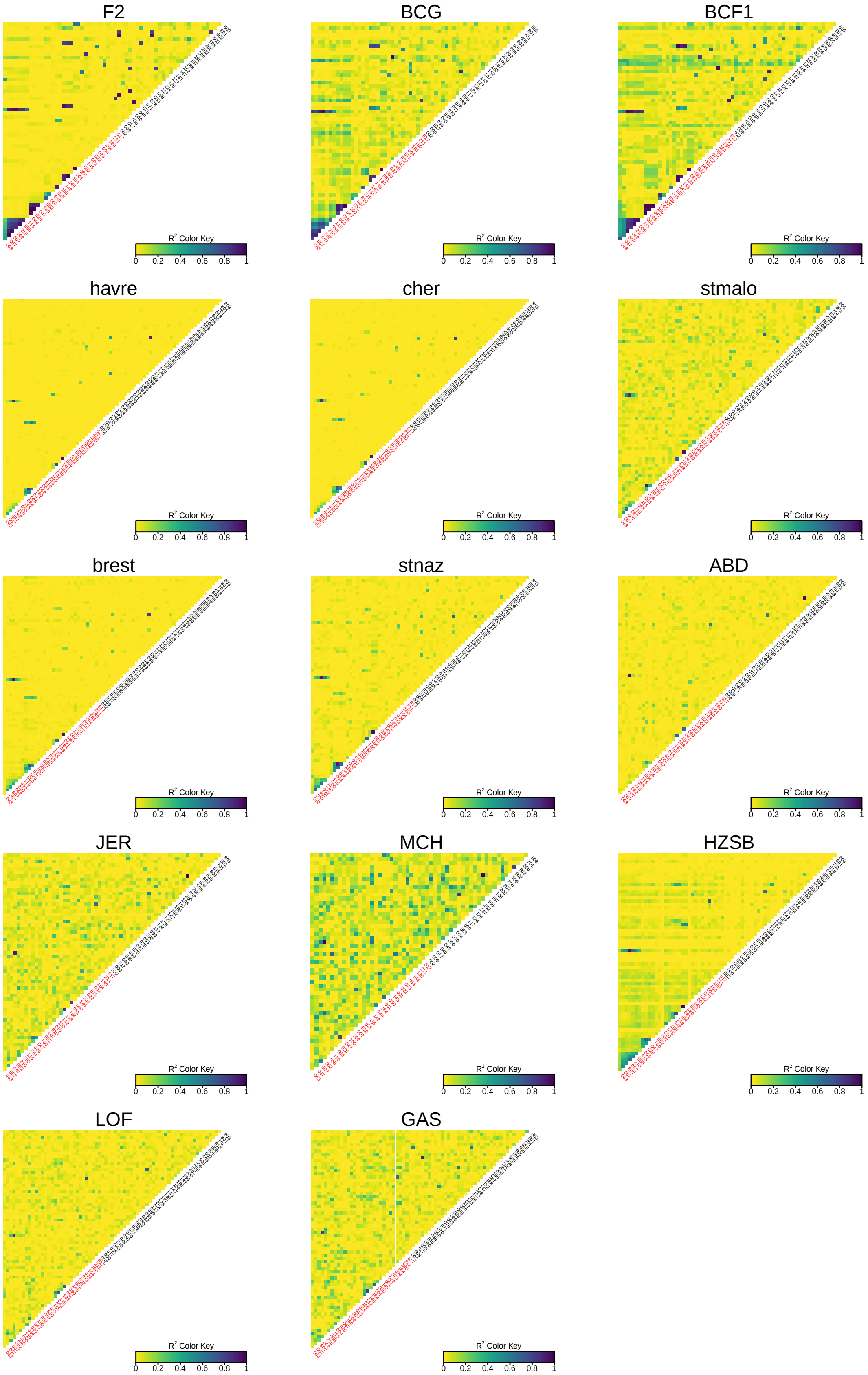

Figure S24: Linkage disequilibrium (R^2) in admixed populations. Markers are order following the genetic map (red labels) and then by name for loci that could be included in the genetic map construction.

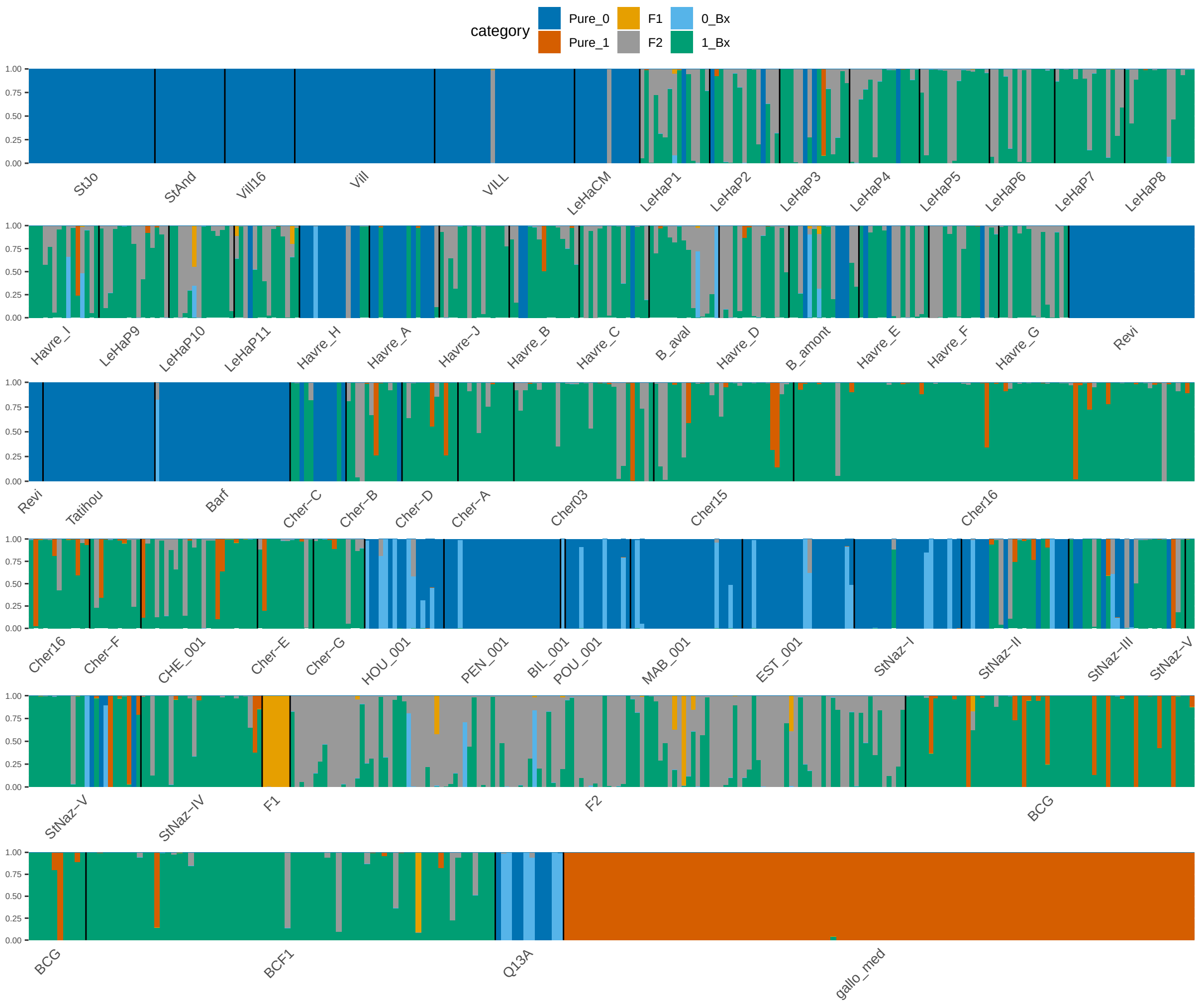

Figure S25: `Newhybrids` analysis including reference Mediterranean *M. galloprovincialis* (Pure_1) and local populations of all ports excluding Brest, containing South-Eu. *M. edulis* individuals (Pure_0). Lab crosses have been included and the results globally conform to the expected genotypes (F1, F2, BCG and BCF1). We hypothesise that pure Med. *M. galloprovincialis* assignements in ports are the result of marker sampling variance, as those assignments are also present in the lab BC samples.

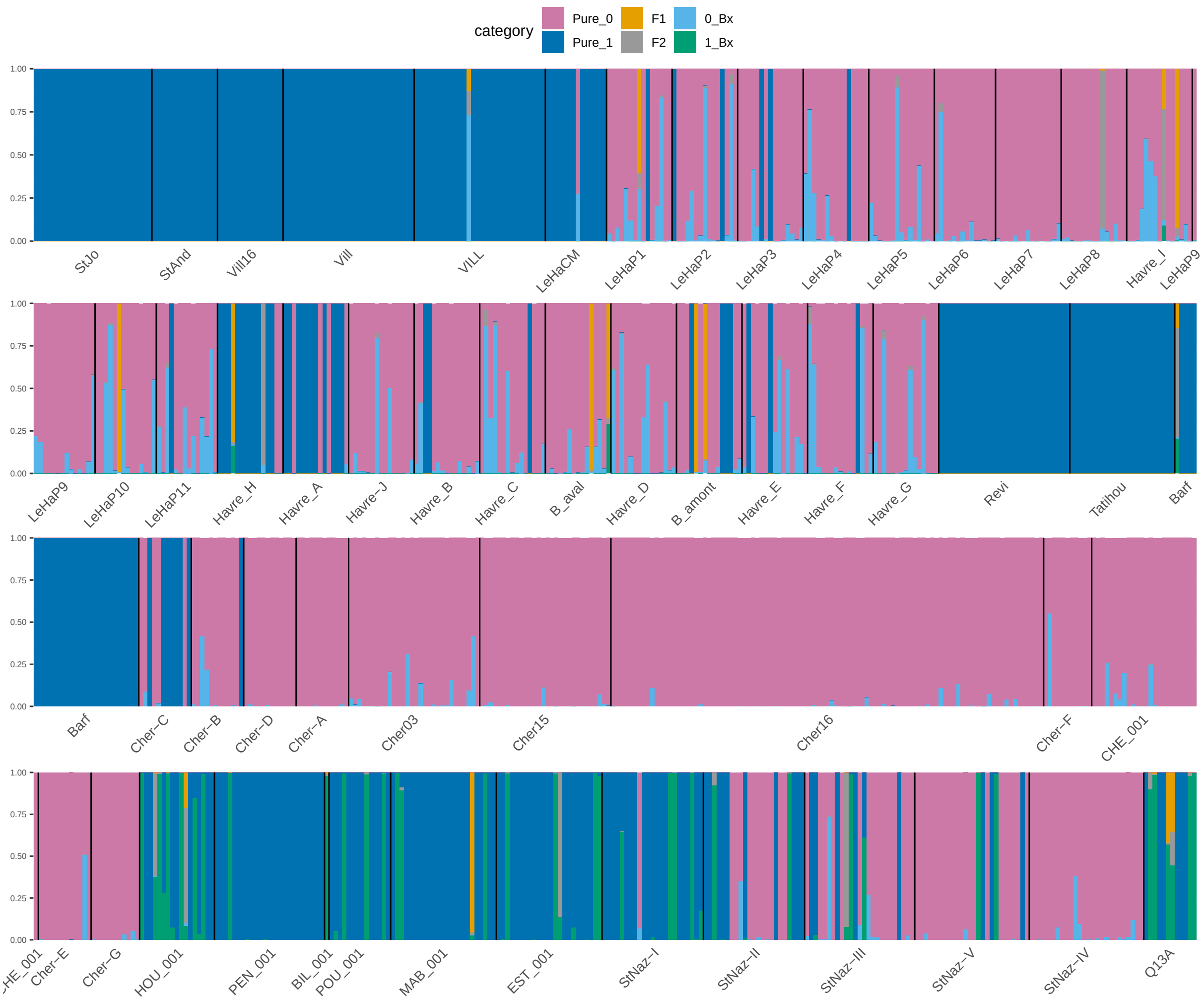

Figure S26: `Newhybrids` analysis comparing dock mussels (Pure_0) and the native Sout-Eu. *M. edulis* (Pure_1). In spite of the filtration to eliminate Atl. *M. galloprovincialis* genotypes, residual ancestry in individuals for populations around Saint-Nazaire (HOU_001 to StNaz-IV) produces spurious 1_Bx assignements.

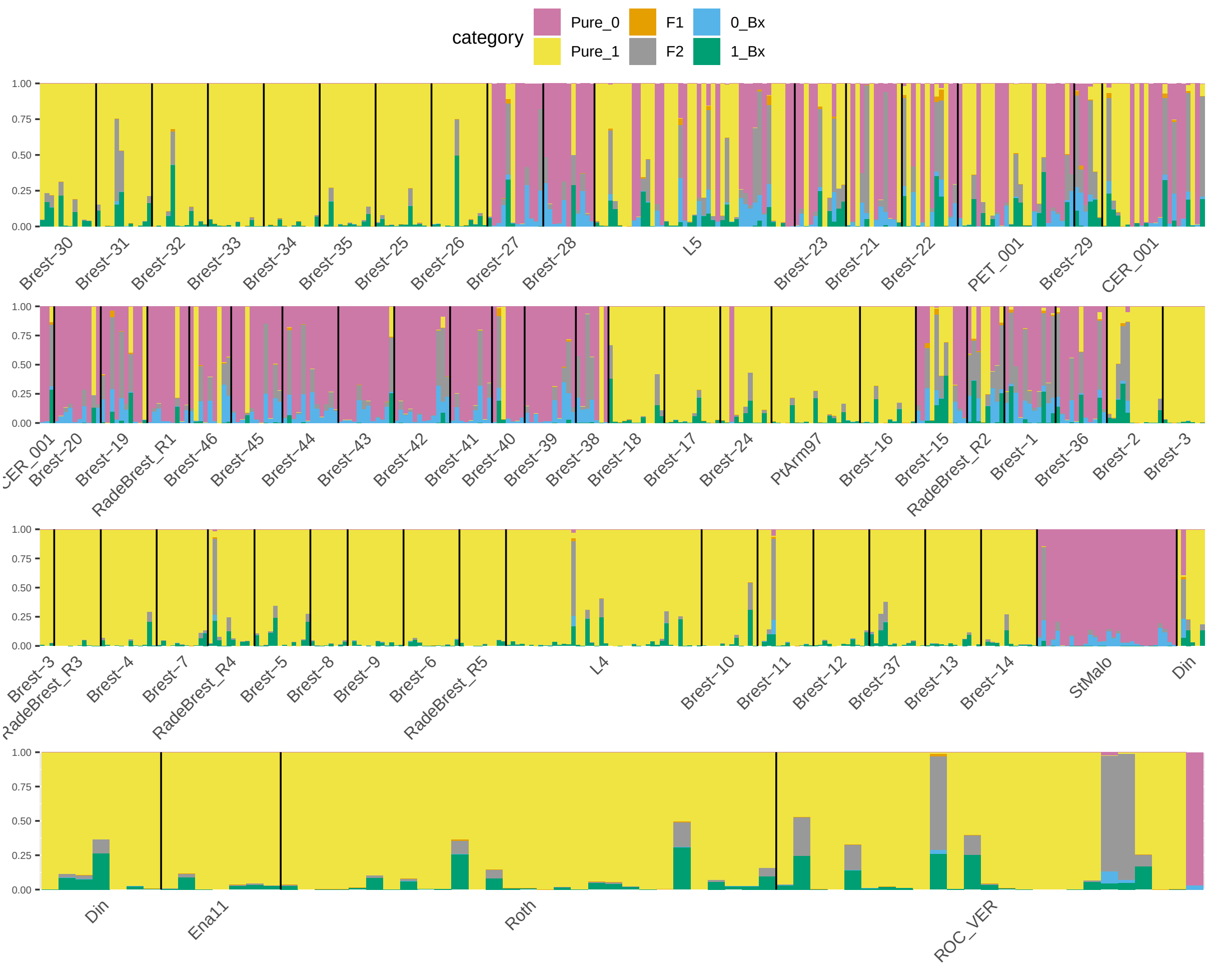

Figure S27: `Newhybrids` analysis comparing dock mussels (Pure_0) and native Atl. *M. galloprovincialis* (Pure_1).

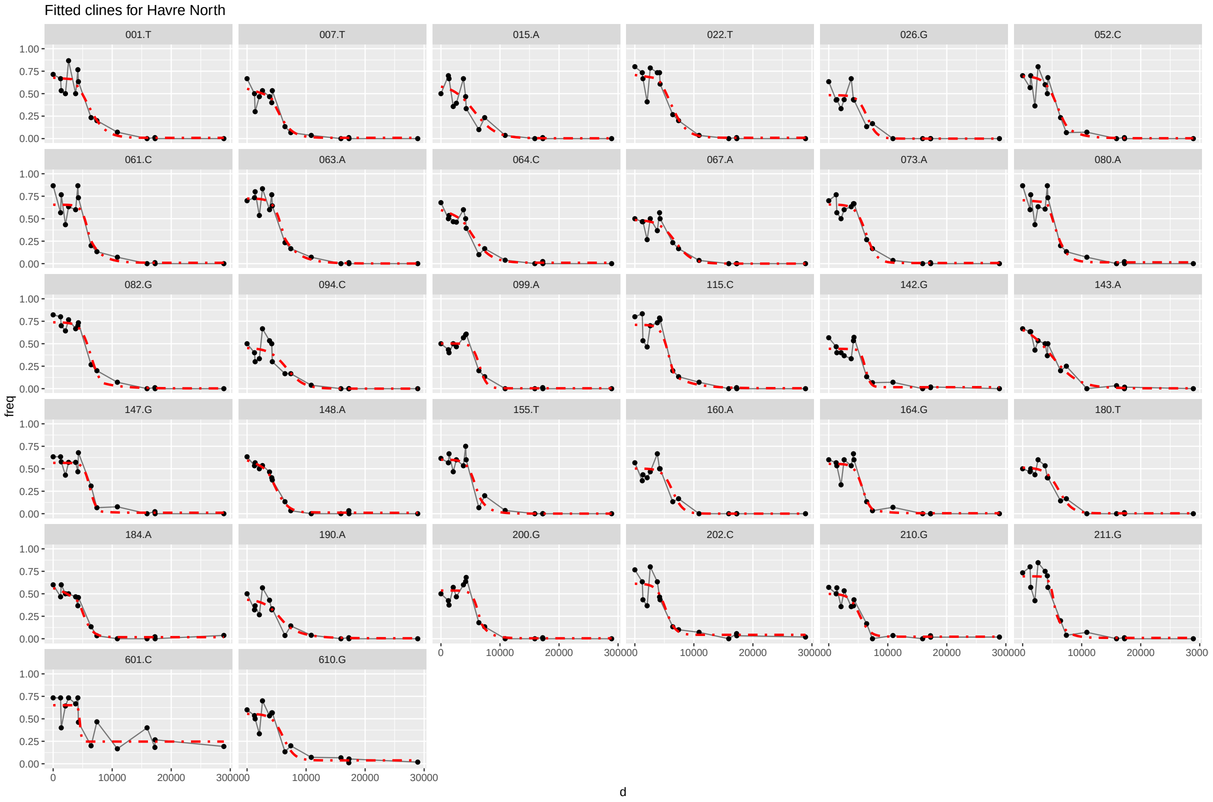

Figure S28: Details of geopgraphic clines per marker fitted in Le Havre North transect. Each point is the allele frequency of the Med. *M. galloprovincialis* allele at one site.

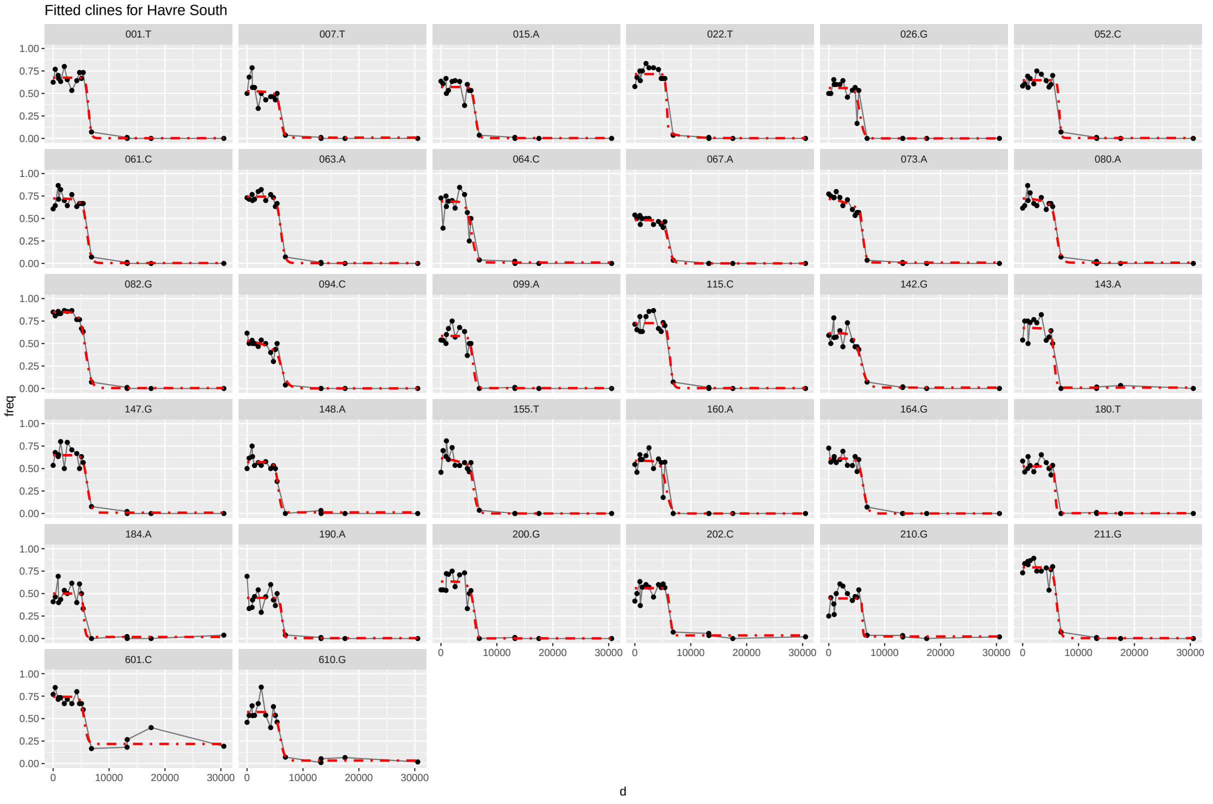

Figure S29: Details of geopgraphic clines per marker fitted in Le Havre South transect.

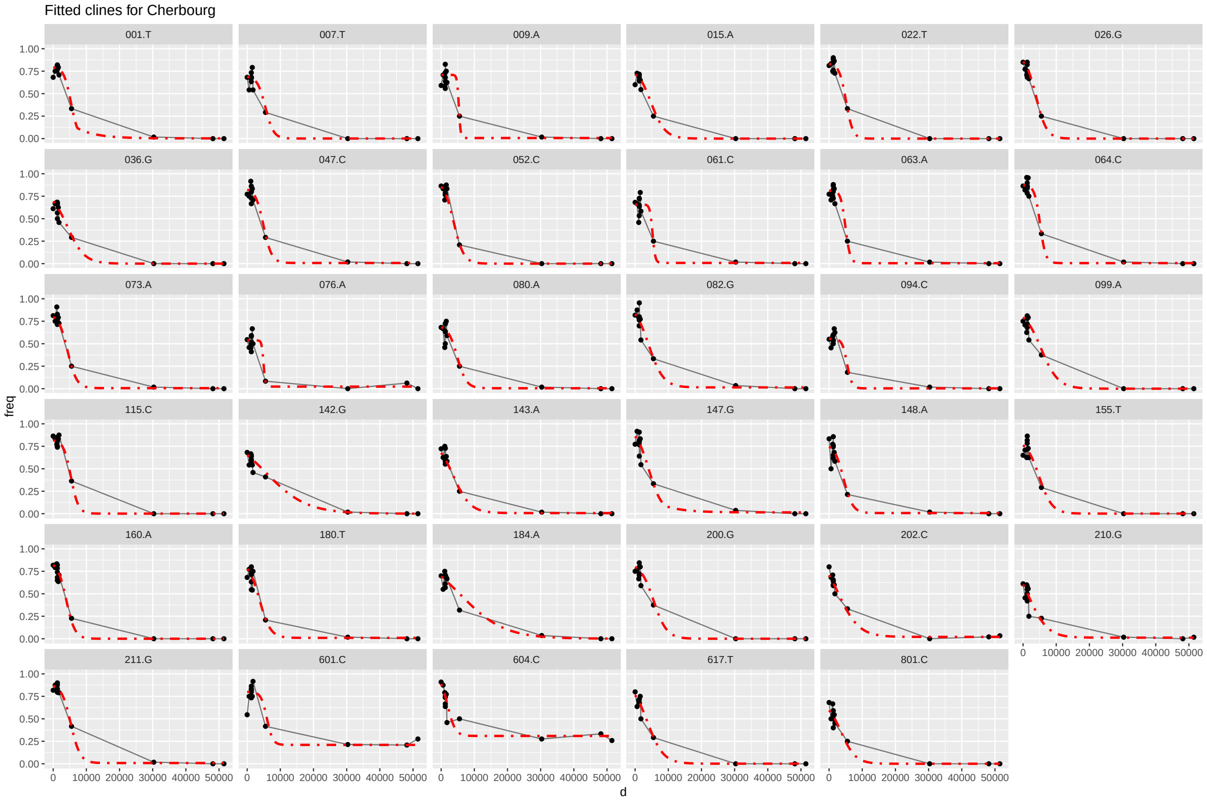

Figure S30: Details of geopgraphic clines per marker fitted in Cherbourg.

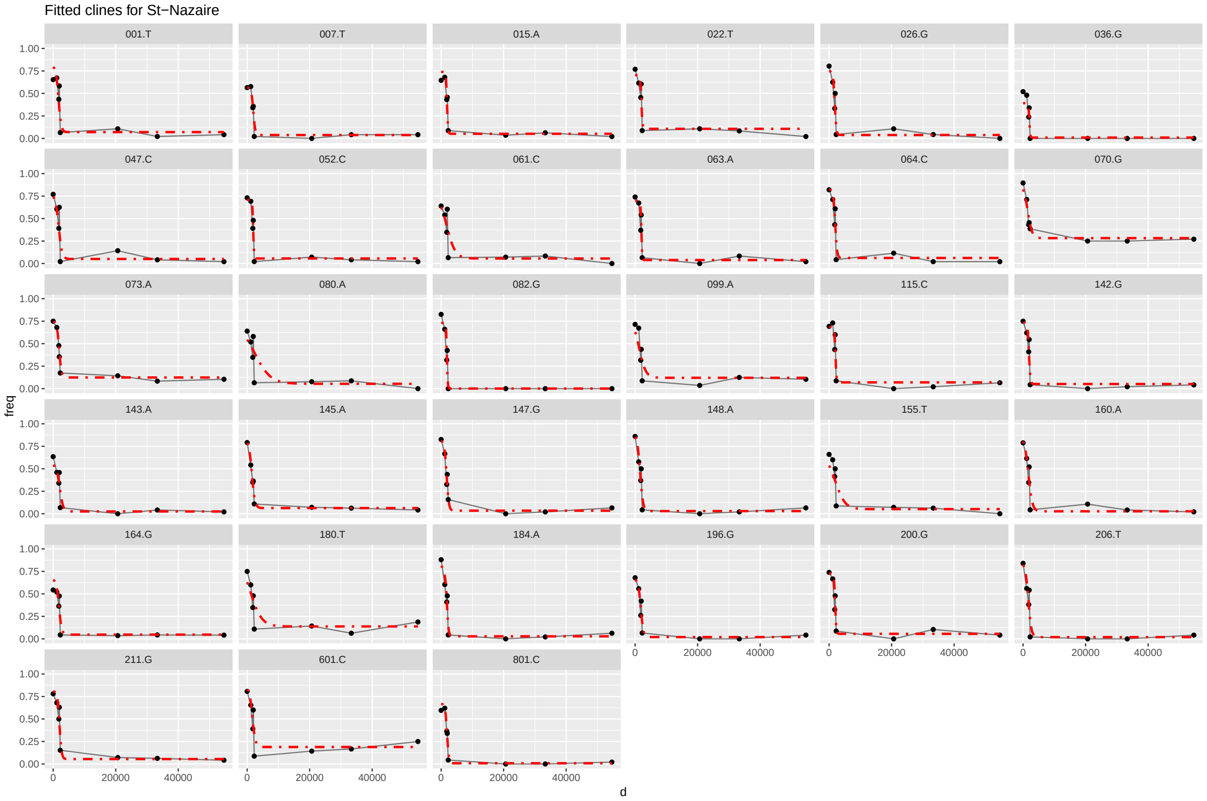

Figure S31: Details of geopgraphic clines per marker fitted in Saint-Nazaire.

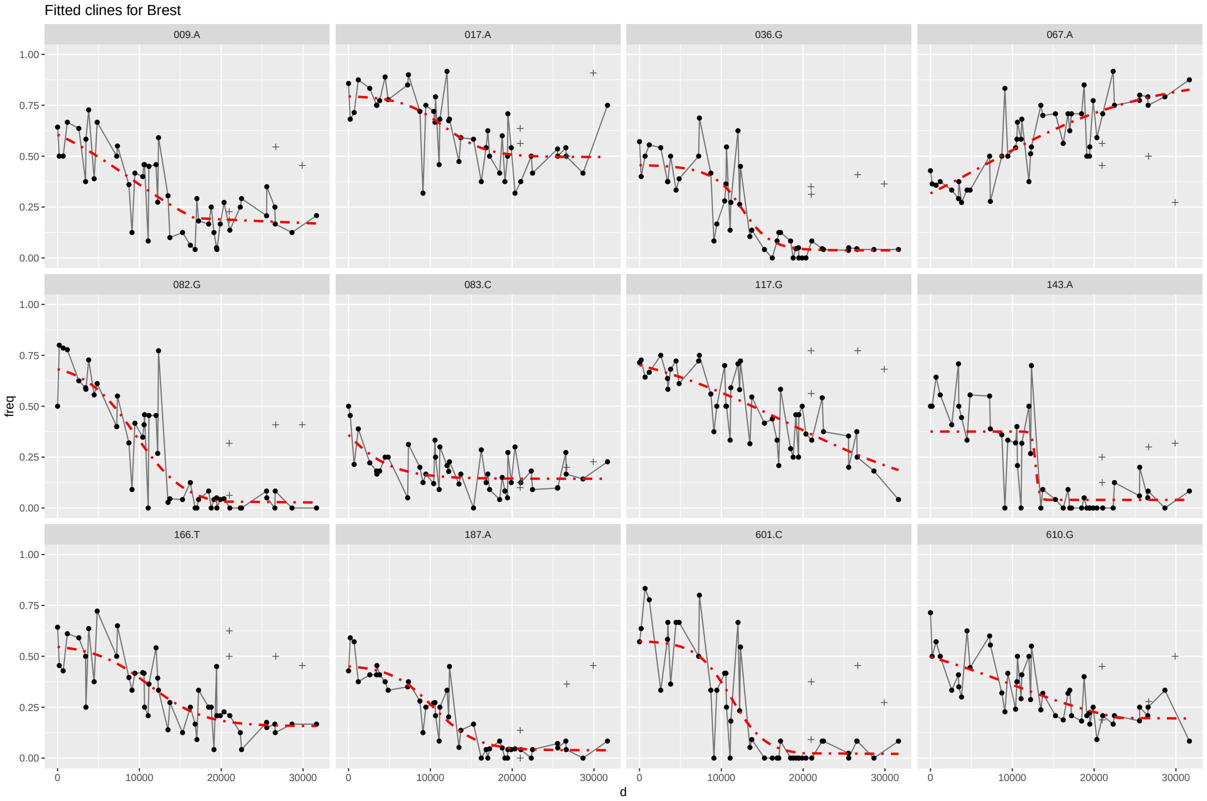

Figure S32: Details of geopgraphic clines per marker fitted in the bay of Brest. Crosses are sites not considered for the fit.

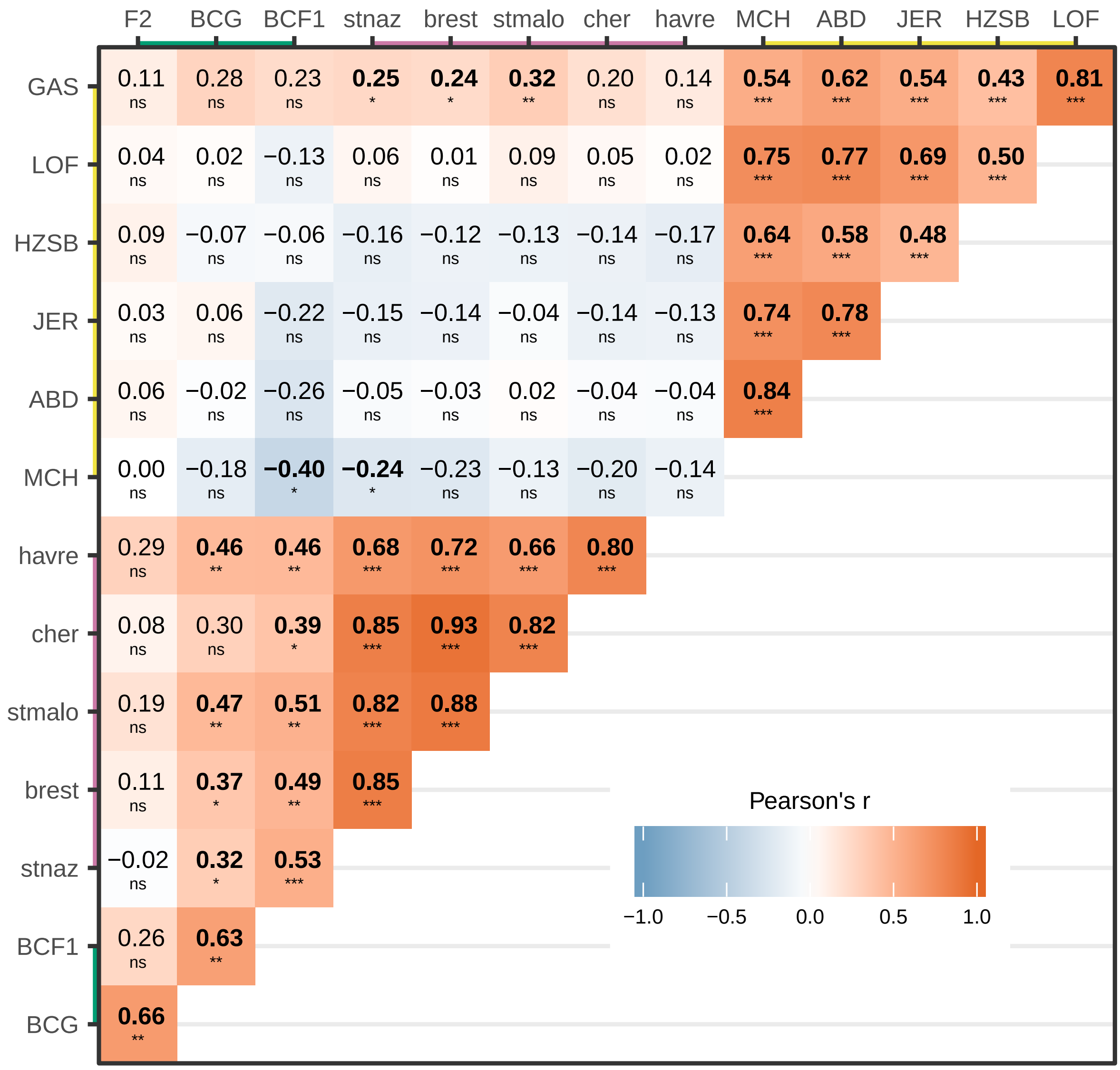

Figure S33: All correlations between populations.

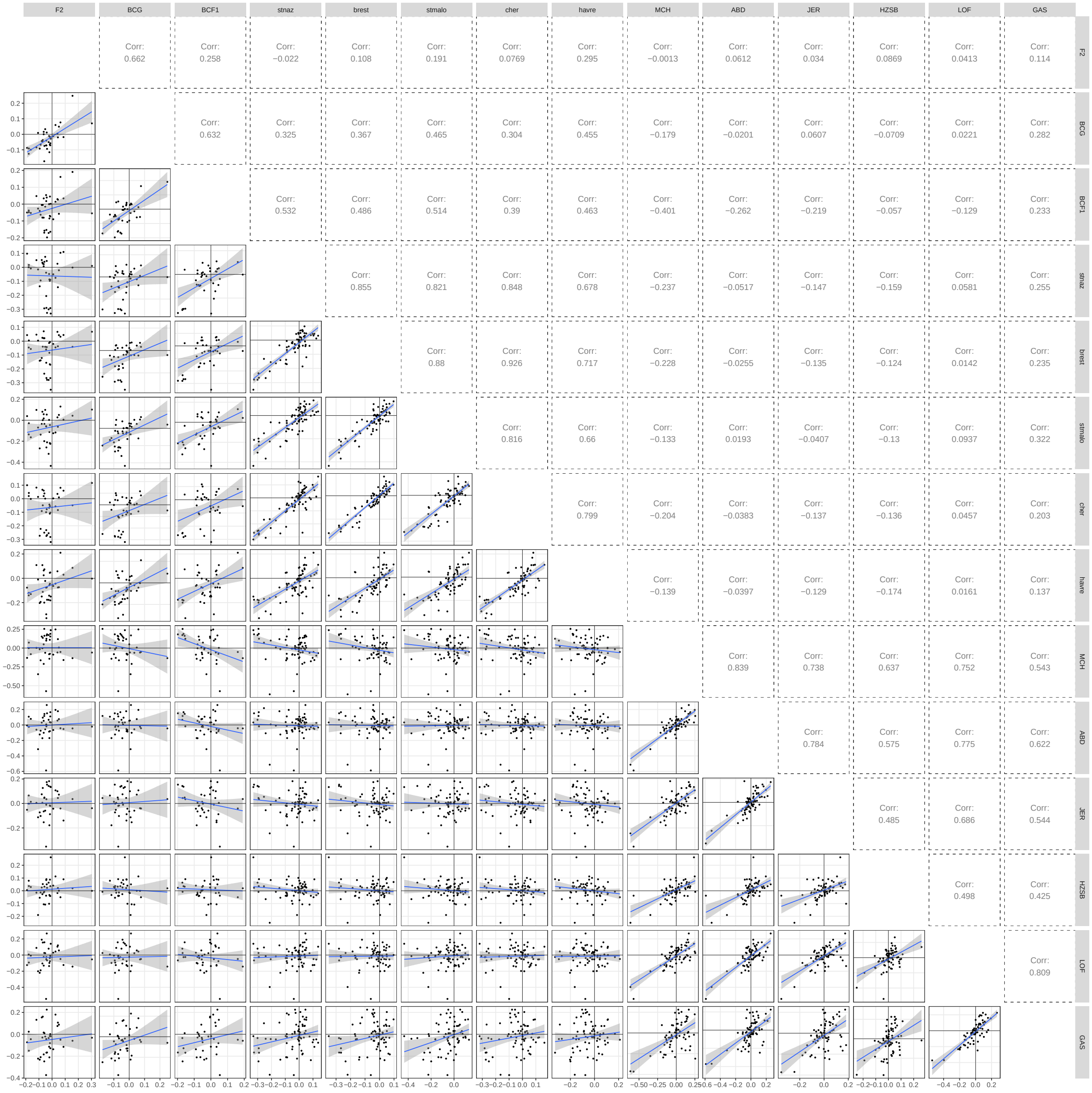

Figure S34: Scatter plots for all correlations between populations.

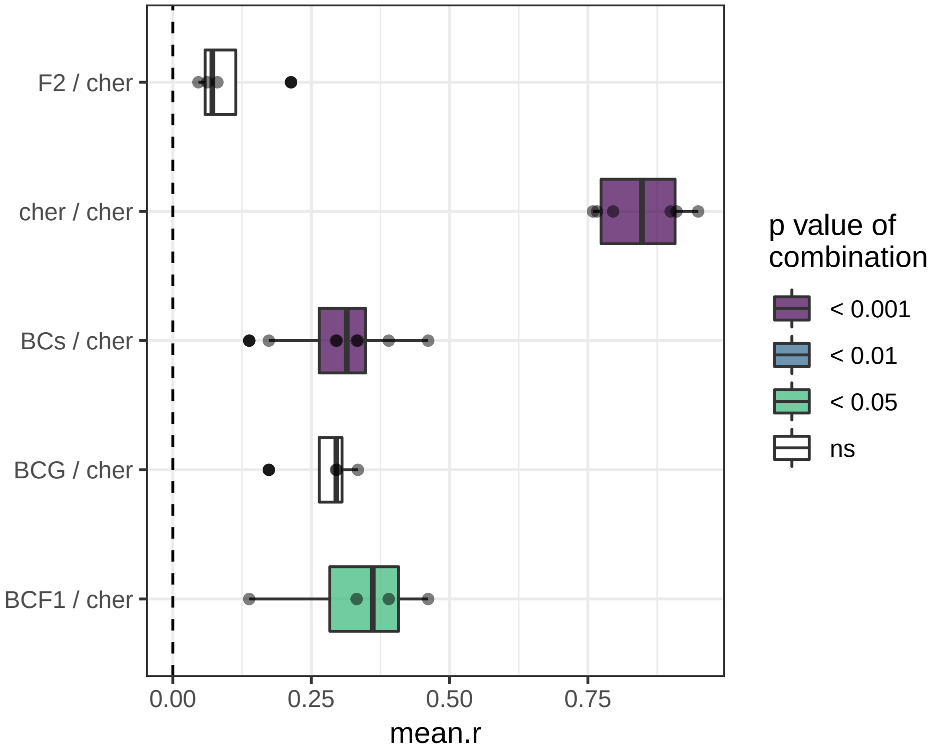

Figure S35: Correlation matrix for temporal variation in Cherbourg.

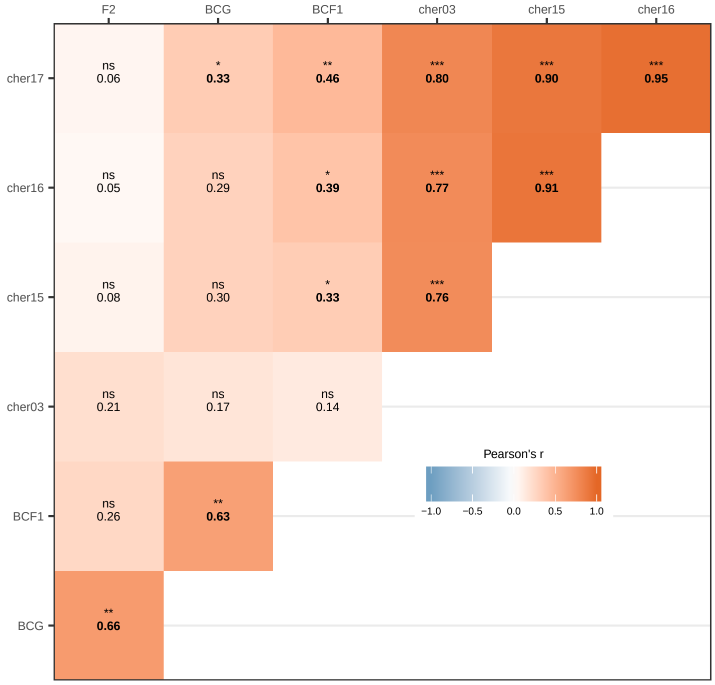

Figure S36: Correlation matrix for temporal variation in Cherbourg.

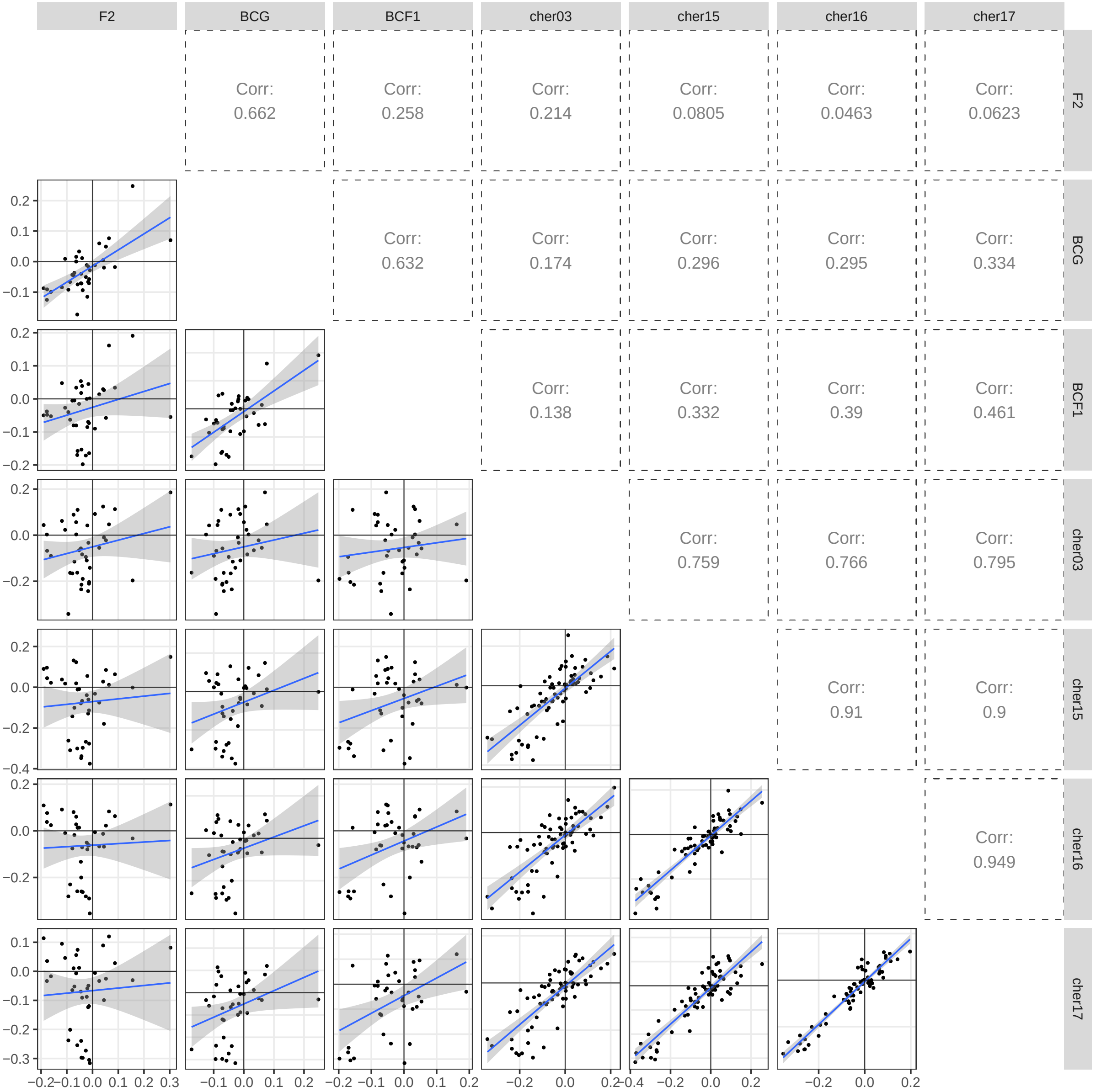

Figure S37: All correlations for Cherbourg.

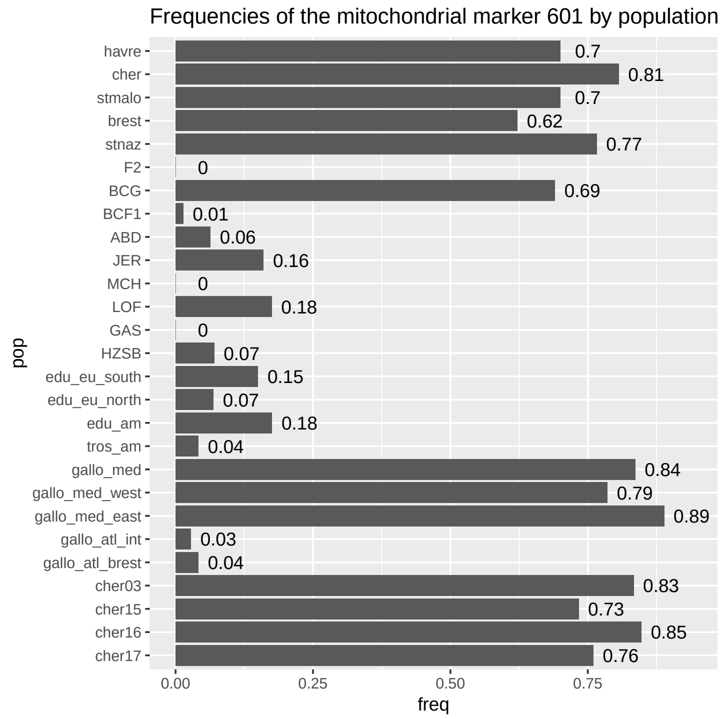

Figure S38: Allele frequencies of the female mitochondrial marker 601.

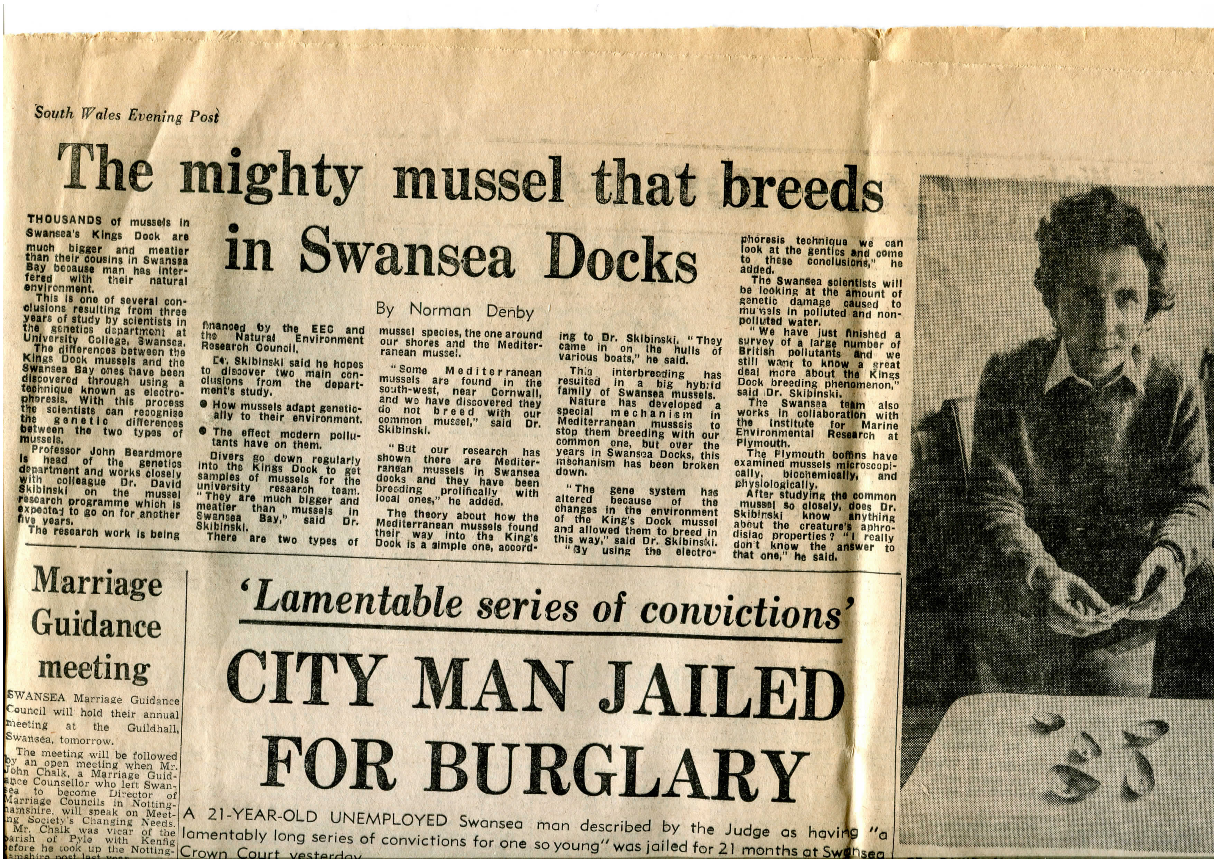

South Wales Evening Post

The mighty mussel that breeds in Swansea Docks

By Norman Denby

THOUSANDS of mussels in Swansea's Kings Dock are much bigger and meatier than their cousins in Swansea Bay because man has interfered with their natural environment.

This is one of several conclusions resulting from three years of study by scientists in the genetics department at University College, Swansea.

The differences between the Kings Dock mussels and the Swansea Bay ones have been discovered through using a technique known as electrophoresis. With this process the scientists can recognise the genetic differences between the two types of mussels.

Professor John Beardmore is head of the genetics department and works closely with colleague Dr. David Skibinski on the mussel research programme which is expected to go on for another five years.

The research work is being financed by the EEC and the Natural Environment Research Council.

Dr. Skibinski said he hopes to discover two main conclusions from the department's study.

- How mussels adapt genetically to their environment.
- The effect modern pollutants have on them.

Divers go down regularly into the Kings Dock to get samples of mussels for the university research team. "They are much bigger and meatier than mussels in Swansea Bay," said Dr. Skibinski.

There are two types of mussel species, the one around our shores and the Mediterranean mussel.

"Some Mediterranean mussels are found in the south-west, near Cornwall, and we have discovered they do not breed with our common mussel," said Dr. Skibinski.

"But our research has shown there are Mediterranean mussels in Swansea docks and they have been breeding prolifically with local ones," he added.

The theory about how the Mediterranean mussels found their way into the King's Dock is a simple one, according to Dr. Skibinski. "They came in on the hulls of various boats," he said.

This interbreeding has resulted in a big hybrid family of Swansea mussels.

Nature has developed a special mechanism in Mediterranean mussels to stop them breeding with our common one, but over the years in Swansea Docks, this mechanism has been broken down.

"The gene system has altered because of the changes in the environment of the King's Dock mussel and allowed them to breed in this way," said Dr. Skibinski.

"By using the electrophoresis technique we can look at the gentics and come to these conclusions," he added.

The Swansea scientists will be looking at the amount of genetic damage caused to mussels in polluted and non-polluted water.

"We have just finished a survey of a large number of British pollutants and we still want to know a great deal more about the Kings Dock breeding phenomenon," said Dr. Skibinski.

The Swansea team also works in collaboration with the Institute for Marine Environmental Research at Plymouth.

The Plymouth boffins have examined mussels microscopically, biochemically, and physiologically.

After studying the common mussel so closely, does Dr. Skibinski know anything about the creature's aphrodisiac properties? "I really don't know the answer to that one," he said.

Marriage Guidance meeting

SWANSEA Marriage Guidance Council will hold their annual meeting at the Guildhall, Swansea, tomorrow.

The meeting will be followed by an open meeting when Mr. John Chalk, a Marriage Guidance Counsellor who left Swansea to become Director of Marriage Councils in Nottinghamshire, will speak on Meeting Society's Changing Needs.

Mr. Chalk was vicar of the parish of Pyle with Kenfig before he took up the Nottinghamshire post last year. [illegible]

'Lamentable series of convictions'

CITY MAN JAILED FOR BURGLARY

A 21-YEAR-OLD UNEMPLOYED Swansea man described by the Judge as having "a lamentably long series of convictions for one so young" was jailed for 21 months at Swansea Crown Court yesterday. [illegible]

Figure S39: The mighty mussels: newspaper article about the Swansea King's dock mussels and the work of Prof. David Skibinski. South Wales Evening Post, 28th of June 1978.

3 Supplementary Tables

Table S1: Population information with sample size after filtration and average missing data among population.

pop	longitude	latitude	locality	date	N	missing data
Aber	-2.07349	57.14758	ABD, Aberdeen, Scotland, UK		74	0.0254
Ault	1.42314	50.09058	Ault, France		36	0.0087
B_amont	0.14587	49.47124	Bassin Amont, Le Havre, France		15	0.0346
B_aval	0.13242	49.47447	Bassin Aval, Le Havre, France		15	0.0165
Barf	-1.25807	49.71015	Barfleur, France	2015	29	0.0152
BCF1			Backcross		72	0.0253
BCG			Backcross		72	0.0130
BDC	1.42282	50.09060	Bois-de-Cise, France	2016	30	0.0056
Berg	5.30573	60.39560	Bergen, Norway		21	0.0235
BFH	-72.46253	41.26056	Brandford Harbor, USA	2009	12	0.0162
BIL_001	-2.49015	47.44528	Le Bile - Penestin, France	11/01/17	25	0.0192
BIL1	-3.08124	43.35194	Zierbena, Bilbao harbor, Spain	17/07/17	12	0.0097
BIL2	-3.07939	43.35262	Zierbena, Bilbao harbor, Spain	17/07/17	12	0.0141
BIL3	-3.03050	43.33065	Santurtzi, Bilbao harbor, Spain	17/07/17	12	0.0097
BIL4	-3.02976	43.33044	Santurtzi, Bilbao harbor, Spain	17/07/17	12	0.0152
BIL5	-3.02063	43.32557	Portugalete, Bilbao harbor, Spain	17/07/17	12	0.0249
BIL6	-3.01302	43.33165	Getxo, Bilbao harbor, Spain	20/07/17	12	0.0119
BIL7	-3.02753	43.34187	Getxo, Bilbao harbor, Spain	20/07/17	12	0.0097
Bizerte	9.86000	37.26000	Bizerte, Tunisia		12	0.0357
BLYT	-1.49830	55.12580	Blythe, North Sea	14/11/14	5	0.0961
Bodo	14.99857	67.21838	Bodø, Norway	16/11/16	38	0.0308
Bost	-71.05000	42.36000	Boston, USA	2009	14	0.0427
Bouf	-0.72468	49.34802	Le Bouffay, France	2015	30	0.0156
Brest-1	-4.28628	48.31669	Lagonna Daoulas, France	08/07/17	12	0.0238
Brest-10	-4.58798	48.27521	Fishing dock, Camaret, France	09/07/17	12	0.0130
Brest-11	-4.59713	48.27972	Camaret marina, France	09/07/17	12	0.0162
Brest-12	-4.61044	48.26152	Veryac'h, France	09/07/17	12	0.0195
Brest-13	-4.49673	48.22357	Morgat marina, France	09/07/17	12	0.0087
Brest-14	-4.46343	48.23806	Postolonnec beach, France	09/07/17	12	0.0087
Brest-15	-4.36913	48.33790	Porz Tinduff, France	24/07/17	12	0.0173

Continued on next page

Table S1 – *Continued from previous page*

pop	longitude	latitude	locality	date	N	missing data
Brest-16	-4.41397	48.33658	Lauberlac'h, France	24/07/17	12	0.0206
Brest-17	-4.44185	48.34370	Anse du Caro, France	24/07/17	12	0.0184
Brest-18	-4.43723	48.35935	Pointe Marloux, France	24/07/17	12	0.0314
Brest-19	-4.43067	48.39313	Marina Moulin Blanc Nord, France	24/07/17	12	0.0130
Brest-2	-4.45367	48.29334	Lanvéoc, France	08/07/17	12	0.0216
Brest-20	-4.43248	48.39002	Marina Moulin Blanc Sud, France	24/07/17	11	0.0519
Brest-21	-4.47392	48.37994	Brest commerce A, France	25/07/17	12	0.0335
Brest-22	-4.47321	48.38280	Brest commerce B, France	25/07/17	12	0.0216
Brest-23	-4.48110	48.37762	Brest commerce C, France	25/07/17	12	0.0325
Brest-24	-4.45348	48.32235	Pointe de l'Armorique, France	25/07/17	11	0.0413
Brest-25	-4.55102	48.35945	Anse de Sainte-Anne, France	25/07/17	12	0.0152
Brest-26	-4.52923	48.36012	Pointe du Porzic Est, France	25/07/17	12	0.0130
Brest-27	-4.49093	48.37732	Marina du Chateau A, France	25/07/17	12	0.0152
Brest-28	-4.48892	48.38018	Marina du Chateau B, France	25/07/17	11	0.0519
Brest-29	-4.45585	48.37470	Brest commerce D, France	25/07/17	12	0.0206
Brest-3	-4.50891	48.28444	Le Fret, France	08/07/17	12	0.0076
Brest-30	-4.78104	48.35904	Le Conquet, France	27/07/17	12	0.0206
Brest-31	-4.77214	48.34305	Penzer, France	27/07/17	12	0.0087
Brest-32	-4.76820	48.32847	Pointe Saint-Mathieu, France	27/07/17	12	0.0108
Brest-33	-4.69895	48.33888	Plougonvelin, France	27/07/17	12	0.0292
Brest-34	-4.61538	48.33758	Petit Minou, France	27/07/17	12	0.0152
Brest-35	-4.58137	48.34809	Le Mengant, France	27/07/17	12	0.0119
Brest-36	-4.26814	48.27982	Térénez, France	05/08/17	12	0.0184
Brest-37	-4.51477	48.20070	île Vierge, France	06/08/17	12	0.0141
Brest-38	-4.34048	48.40575	Elorn 2, France	21/09/17	7	0.0148
Brest-39	-4.34317	48.40582	Elorn 2B, France	21/09/17	12	0.0357
Brest-4	-4.53997	48.29194	L'île du Renard, France	08/07/17	12	0.0065
Brest-40	-4.34898	48.40385	Elorn St Jean, France	21/09/17	11	0.0153
Brest-41	-4.35612	48.40648	Elorn 3, France	21/09/17	9	0.0216
Brest-42	-4.37208	48.39705	Elorn 6, France	21/09/17	12	0.0249
Brest-43	-4.38280	48.39553	Elorn 9, France	21/09/17	12	0.0216
Brest-44	-4.38205	48.39300	Elorn 11, France	21/09/17	12	0.0238
Brest-45	-4.38670	48.39298	Elorn 14, France	21/09/17	12	0.0173
Brest-46	-4.39465	48.39028	Elorn 15, France	21/09/17	12	0.0184

Continued on next page

Table S1 – *Continued from previous page*

pop	longitude	latitude	locality	date	N	missing data
Brest-5	-4.54507	48.31460	Roscanvel, France	08/07/17	12	0.0249
Brest-6	-4.57300	48.30710	La Fraternité, France	08/07/17	12	0.0173
Brest-7	-4.55452	48.29658	Quelern, France	09/07/17	12	0.0141
Brest-8	-4.53207	48.33974	Pointe des Espagnols, France	09/07/17	8	0.0568
Brest-9	-4.58254	48.31834	Îlot des Capucins, France	09/07/17	12	0.0141
BretN__g1	-3.71707	48.70014	venizella, Guimaec, France		10	0.0104
BretN__g2	-3.69819	48.69000	poul roudou, Locquirec, France		10	0.0078
BretN__g3	-3.67690	48.68796	lezingar, Locquirec, France		10	0.0104
BretN__g4	-3.64643	48.69481	pors ar villiec, Locquirec		10	0.0052
BretN__i1	-3.54638	48.72863	Léguer estuary, South point, France		9	0.0072
BretN__i2	-3.54230	48.72895	Banc du Guer sud, France		10	0.0117
BretN__i3	-3.58156	48.72139	Locquémo, France		10	0.0078
BretN__i4	-3.58371	48.72608	Locquemo, France		10	0.0065
BretN__i5q1	-3.58283	48.68886	Beg ar form, Saint-Michel en Grève, France		10	0.0091
BretN__i5q2	-3.58271	48.68914	Beg ar form, Saint-Michel en Grève, France		10	0.0039
BretN__i6	-3.61713	48.68507	Beg Douar, Plestin Les Grèves, France		10	0.0078
BretN__i8	-3.80425	48.72304	Roc'h Goalen, Plougasnou, France		10	0.0312
CAD	-6.29842	36.52719	Cadix, Spain	2016	15	0.0294
Can	3.69184	43.41226	Sète, canal, France		3	0.0909
Cart	-1.80842	49.37165	Carteret, France	2015	29	0.0331
CER__001	-4.44887	48.38265	Brest port, France	14/12/16	25	0.0135
Cha	-1.08059	46.03229	Charente, France		12	0.0119
CHE	-2.29965	47.23232	Pointe de Chemoulin, France	2016	30	0.0242
CHE__001	-1.62336	49.64574	Cherbourg, France	02/12/16	25	0.0156
Cher-A	-1.61953	49.64530	Cherbourg A, France	01/08/17	12	0.0227
Cher-B	-1.61981	49.64956	Cherbourg B, France	01/08/17	12	0.0249
Cher-C	-1.63244	49.67307	Cherbourg C, France	01/08/17	12	0.0390
Cher-D	-1.62020	49.64818	Cherbourg D, France	01/08/17	12	0.0487
Cher-E	-1.62080	49.64208	Cherbourg E, France	01/08/17	12	0.0433
Cher-F	-1.62342	49.64566	Cherbourg F, France	01/08/17	11	0.0342
Cher-G	-1.61964	49.63846	Cherbourg G, France	01/08/17	11	0.0614
Cher03	-1.61994	49.63942	Cherbourg, France		30	0.0108
Cher15	-1.62332	49.64557	Cherbourg, France	2015	30	0.0212
Cher16	-1.62332	49.64557	Cherbourg, France	2016	99	0.0365

Continued on next page

Table S1 – *Continued from previous page*

pop	longitude	latitude	locality	date	N	missing data
CHR	-71.51917	41.38692	Charlestown, Snug Harbor Marina, USA	2009	12	0.0206
COR	-8.41393	43.37011	Coruña, Galicia, Spain	2016	15	0.0069
CRE	-8.97390	38.48047	Praia do Creio, Arrábida, Spain	2016	15	0.0078
Croa	13.62091	45.07155	Croatia		14	0.0547
D15A	2.93460	54.32470	D15-A, North Sea	03/10/15	8	0.1445
Dieppe	1.09670	49.93556	Dieppe, France		48	0.0095
Din	-2.04937	48.63913	Dinard, France		13	0.0649
DKL	-15.93000	23.72000	Dahkla, Morocco		10	0.1013
Dun	2.42178	51.10313	Dunkerque, France	2016	30	0.0030
Ena11	-2.07507	48.64035	Saint-Enogat, Dinard, France		7	0.0816
Engl	-0.93377	49.39478	Englesqueville, France	2015	30	0.0177
EST_001	-2.53370	47.30370	Le Croisic, France		25	0.0088
F1			F1		6	0.0195
F2			F2		132	0.0364
Far	-7.93884	37.01568	Faro, Portugal		8	0.0633
FAR	-7.93884	37.01568	Faro, Portugal	2016	15	0.0190
Fer11	-3.96309	48.72045	ballast ferry	2011	38	0.0123
Fer13	-3.96309	48.72045	ballast ferry Armorique	16/05/13	27	0.0077
FINO	7.15830	55.19500	FINO 3, North Sea	23/09/15	6	0.2186
GA	6.24164	62.46399	GAS, Gaseid, Alesund, Norway	2015	36	0.0400
Gdansk	18.52794	54.60956	Gulf of Gdansk, Poland	23/08/12	12	0.1223
Gran	-1.59947	48.84003	Granville, France	2015	31	0.0201
GrCa	-1.03633	49.40803	Grancamp, France	2015	29	0.0233
Groix	-3.45911	47.64701	Groix, France		48	0.0249
HARW	1.28130	51.93480	Harwich, North Sea	15/11/15	11	0.1181
Havre_A	0.11327	49.48586	Le Havre, France	2016	15	0.0069
Havre_B	0.11612	49.47316	Le Havre, France	2016	15	0.0303
Havre_C	0.12268	49.47000	Le Havre, France	2016	15	0.0130
Havre_D	0.13867	49.46841	Le Havre, France	2016	15	0.0139
Havre_E	0.15604	49.46831	Le Havre, France	2016	15	0.0104
Havre_F	0.15841	49.47585	Le Havre, France	2016	15	0.0095
Havre_G	0.17341	49.47242	Le Havre, France	2016	15	0.0087
Havre_H	0.09767	49.48713	Le Havre, France	2016	15	0.0052
Havre_I	0.16510	49.45401	Le Havre, France	2016	15	0.0173

Continued on next page

Table S1 – *Continued from previous page*

pop	longitude	latitude	locality	date	N	missing data
Havre-J	0.11832	49.48893	Le Havre, France	02/07/17	15	0.0502
Her	25.14420	35.33800	Heraklion, Greece		35	0.0171
Holl	5.42400	53.31000	Wadden Sea, The Netherlands		23	0.0056
HORN	7.81100	55.47890	Horns Rev, North Sea	10/06/15	2	0.1299
HOU_001	-2.93723	47.41967	Ile de Houat, France	10/01/17	25	0.0192
JapSea	135.09238	38.73579	Katolikova, Japan Sea		16	0.1631
Jer	-2.02038	49.17308	JER, Jersey, France	2016	30	0.0143
JUM	-3.90044	47.83397	La Jument, France	2016	30	0.0104
K10B	3.25390	53.36260	K10-B, North Sea	01/10/14	8	0.1591
KOR	128.44707	34.83393	South Korea		10	0.1169
L4	-4.57230	48.28220	bay of Brest (Camaret), France	24/04/17	42	0.0247
L5	-4.48815	48.37733	bay of Brest (port), France	24/04/17	49	0.0358
LaRochA	-1.22359	46.14988	La Rochelle, France	24/06/17	7	0.1410
LaRochB	-1.22449	46.15045	La Rochelle, France	25/06/17	29	0.0636
LeHaCM	0.11380	49.45725	Le Havre, France	2017	14	0.0241
LeHaP1	0.10579	49.46466	Le Havre P1, France	2017	15	0.0277
LeHaP10	0.17332	49.45352	Le Havre P10, France	2017	14	0.0473
LeHaP11	0.17807	49.45456	Le Havre P11, France	2017	15	0.0857
LeHaP2	0.10996	49.46040	Le Havre P2, France	2017	15	0.0199
LeHaP3	0.11473	49.45825	Le Havre P3, France	2017	15	0.0156
LeHaP4	0.12137	49.45734	Le Havre P4, France	2017	15	0.0216
LeHaP5	0.13330	49.45634	Le Havre P5, France	2017	15	0.0727
LeHaP6	0.14413	49.45546	Le Havre P6, France	2017	15	0.0355
LeHaP7	0.15224	49.45185	Le Havre P7, France	2017	15	0.0268
LeHaP8	0.16015	49.45441	Le Havre P8, France	2017	15	0.0268
LeHaP9	0.16602	49.45376	Le Havre P9, France	2017	15	0.0502
LISB	-9.09260	38.76350	Lisbon, Portugal	14/02/15	5	0.1818
LochEti	-5.18317	56.45735	Loch Etive, Scotland, UK		11	0.1145
Locq	-3.59720	48.73691	Locquemeau, France		37	0.0190
LOF	13.87800	68.33800	Lofoten, Norway	2014	43	0.0417
MAB_001	-2.21610	46.99760	Maison Blanche, Noirmoutier, France		25	0.0088
MAK	22.61232	40.41618	Makrigiallos, Greece		6	0.1017
MOU	-4.04187	47.84282	Pointe de Mousterlin, France	2016	30	0.0242
Murch	1.75000	61.40000	MCH, Murchison oil station, Norwegian Sea		12	0.0465

Continued on next page

Table S1 – *Continued from previous page*

pop	longitude	latitude	locality	date	N	missing data
NUS	-51.71040	64.19680	Nuuk, Greenland	2014	28	0.0756
NY-harb-A	-73.79327	40.79521	NYC Throgs Neck Bridge, USA	09/06/04	9	0.0534
NY-harb-B	-71.94682	41.02913	Montauk, USA	23/02/14	7	0.0686
NY-harb-C	-74.01726	40.65532	Brooklyn piers, USA		10	0.0260
ORT	-7.80508	43.72246	Ortigueira, Galicia, Spain	2016	15	0.0139
OST	-72.38419	41.26328	Old Saybrook town, Hartland drive, USA	2009	24	0.0211
Ostende	2.91815	51.23815	Ostende, Belgium		29	0.0090
Ouis	-0.45892	49.33829	Ouistreham, France	2015	30	0.0203
Palice-A	-1.21576	46.15909	La Palice, Bassin à flot, France	23/02/18	18	0.0065
Palice-B	-1.21843	46.15854	La Palice, lock, France	23/02/18	76	0.0106
PEN	-2.51383	47.30632	Barres de Pen Bron, France	2016	30	0.0212
PEN_001	-2.49363	47.49712	Camaret plage-Penestin, France	11/01/17	25	0.0223
Peniche	-9.37944	39.36844	Peniche, Portugal	2016	15	0.0121
PET_001	-4.47146	48.38119	Brest port, France	14/12/16	25	0.0151
Pir	-1.60083	49.16588	Pirou, France	2016	30	0.0074
POU_001	-2.41596	47.26029	Le Pouliguen - La Baule, France	12/01/17	25	0.0119
Prim	-3.82080	48.72122	Primel, France		36	0.0177
PtArm97	-4.45355	48.32563	Pointe de l'Armorique, plage des ducs d'albes, France		19	0.0444
Q13A	4.13610	52.19110	Q13-A, North Sea	28/05/14	12	0.0617
QAS	-69.24030	77.46500	Qaanaaq, Greenland	2014	30	0.1364
RadeBrest_R1	-4.39973	48.39014	Pont d'Iroise, France		10	0.0195
RadeBrest_R2	-4.36882	48.33810	Port du Tinduff, France		10	0.0169
RadeBrest_R3	-4.50680	48.28679	Le Fret, France		10	0.0091
RadeBrest_R4	-4.55083	48.30609	Quélern, Roscanvel, France		10	0.0104
RadeBrest_R5	-4.57194	48.28199	Pte Ste Barbe, Camaret, France		10	0.0104
Rave	-1.21023	49.47333	Ravenoville, France	2015	30	0.0130
Revi	-1.22933	49.57467	Réville, France	2015	30	0.0160
ROC_VER	-1.40556	45.98358	Rocher Vert Chaucre cote ouest Oléron, France	05/10/16	25	0.0151
RoRo	-3.38314	48.81866	Roc-Rouge, France		32	0.0114
Roth	-1.96213	48.68703	Rothéneuf, France		32	0.0288
SB1	-2.71614	48.55568	Pointe du Roselier, France		10	0.0039
SB2	-2.78885	48.58458	Pordic, plage du petit havre, France		10	0.0052
SCHV	4.25820	52.09870	Scheveningen, North Sea	08/07/14	1	0.0779
SoRo	-1.55248	48.73239	Sol-Roc, France		30	0.0472

Continued on next page

Table S1 – *Continued from previous page*

pop	longitude	latitude	locality	date	N	missing data
StAnd	0.07980	49.54744	Saint-Andrieux, France		15	0.0087
StJo	0.15264	49.64395	Saint-Jouin, France	2015	27	0.0106
StLau-CBD	-69.46615	48.26941	Saint Lawrence, Cap de Bon Désir, Canada		9	0.1010
StLau-TD	-69.69614	48.13441	Saint Lawrence, Tadoussac, Canada		12	0.1180
StMalo	-2.02165	48.64855	Saint-Malo port, France	16/07/17	30	0.0156
StNaz-I	-2.19894	47.27074	Saint-Nazaire, France	30/01/18	26	0.0080
StNaz-II	-2.20027	47.27391	Saint-Nazaire, France	30/01/18	25	0.0348
StNaz-III	-2.20259	47.27312	Saint-Nazaire, France	30/01/18	25	0.0358
StNaz-IV	-2.19813	47.28964	Saint-Nazaire, France	30/01/18	26	0.0330
StNaz-V	-2.19880	47.27941	Saint-Nazaire, France	30/01/18	26	0.0280
SV1	11.13620	79.11230	Svalbard – Kongsfjorden, Norway	2012	6	0.0455
SV2	11.13620	79.11230	Svalbard – Kongsfjorden, Norway	2013	9	0.0505
SV3	11.13620	79.11230	Svalbard – Kongsfjorden, Norway	2014	12	0.0649
SV4	15.60260	78.23810	Svalbard – Adventfjorden, Norway	2014	11	0.0106
Tatihou	-1.23627	49.58673	Tatihou, France	09/07/05	24	0.0076
Th16	3.69179	43.41227	Thau, France	2016	23	0.0169
TH16B	3.68782	43.41474	Thau, ponton station, France	2016	18	0.0043
VHO	-8.82312	37.38853	Vale dos Homens, Aljezur, Spain	2016	15	0.0078
VIG	-8.70214	42.26008	Vigo, Spain	2016	15	0.0104
Vill	0.11605	49.39935	Villerville, France	2015	30	0.0251
VILL	0.12383	49.40374	Villerville, France	2016	30	0.0108
Vill16	0.12351	49.40369	Villerville, France	2016	15	0.0035
VLR	0.79465	49.87812	Veules-les-Roses, France	2016	30	0.0052
Wim	1.60307	50.77263	Wimereux, France	2016	29	0.0049

Continued on next page

Table S2: Softwares and R packages used in this study

**software**	**version**	**reference**	**link**
R	3.5.2	R Core Team, 2019	https://www.r-project.org/
Python	3.7.1	-	https://www.python.org/
Structure	2.3.4	Falush et al., 2003; Pritchard, Stephens, and Donnelly, 2000	https://web.stanford.edu/group/pritchardlab/structure.html
Admixture	1.3.0	Alexander and Lange, 2011	http://software.genetics.ucla.edu/admixture/
CLUMPAK	1.1	Kopelman, Mayzel, Jakobsson, Rosenberg, and Mayrose, 2015	http://clumpak.tau.ac.il/
QGIS	3.4.2	-	https://qgis.org/fr/site/
Newhybrids	1.1 beta 3	Anderson and Thompson, 2002	http://ib.berkeley.edu/labs/slatkin/eriq/software/software.htm

**R package**	**version**	**reference**
tidyverse	1.2.1	Wickham, 2017
reshape2	1.4.3	Wickham, 2007
adegenet	2.1.1	T. Jombart and Ahmed, 2011; Thibaut Jombart, 2008
pegas	0.11	Paradis, 2010
hierfstat	0.04-22	Goudet and Jombart, 2015
introgress	1.2.3	Gompert and Buerkle, 2010
qtl	1.44-9	Broman, Wu, Sen, and Churchill, 2003
LDheatmap	0.99-5	Shin, Blay, McNeney, and Graham, 2006
genetics	1.3.8.1	Warnes, Gorjanc, Leisch, and Man, 2013
hzar	0.2-5	Derryberry, Derryberry, Maley, and Brumfield, 2014
ggpubr	0.2	Kassambara, 2018
psych	1.8.12	Revelle, 2018
EmpiricalBrownsMethod	1.10.0	Poole, Gibbs, Shmulevich, Bernard, and Knijnenburg, 2016
ggstatsplots	0.0.9	Patil, 2019
foreach	1.4.4	Microsoft and Weston, 2017
doMC	1.3.5	Analytics and Weston, 2017
gdistance	1.2-2	van Etten, 2017

Table S3: Across all analyses multiple selection threshold have been used to select either individuals or markers for specific analyses. Decision thresholds are presented in table S3. For statistical tests we chose $\alpha = 0.05$ as the significant threshold.

Variable used	for what?	with what?	kept if
Missing data of individuals	full dataset	genotypes	$< 30\%$
	genetic map F2	genotypes	$< 10\%$
Missing data of markers	full dataset	genotypes	$< 10\%$
	F_{ST} between reference populations	genotypes of *M. trossulus* pop. only	$< 30\%$
Q value or prob. of membership (C)	reference groups filtration	`Admixture` $K = 3$ (fig. S4)	$Q > 85\%$ from putative cluster
	M. trossulus individuals	full `Structure` $K = 5$ (fig. S5)	$Q_{tros} > 0.1$
	list of dock mussels	`Structure` local w/o admixture $K = 3$ (fig. S20)	$\in$ (Port) $\wedge \in (C_{dock} > 0.9)$
	list of *M. edulis* in ports	`Structure` local w/o admixture $K = 3$	$\in$ (Port) $\wedge \in (C_{edu} > 0.5)$
	list of gallo_atl in ports	`Structure` local w/o admixture $K = 3$	$\in$ (Port) $\wedge \in (C_{gallo\_atl} > 0.5$
	list of admix gallo_atl	`Structure` w/o admixture $K = 5$ (fig. S19)	$\in$ (focal pop.) $\wedge \in (C_{gallo\_atl} + C_{admix\_atl} > 0.9)$

Table S4: Reference panel groups and populations

L1	L2	L3	pop	N
edu	edu_am	edu_am	Bost	14
			CHR	12
			OST	24
	edu_eu	edu_eu_south	Dun (ext)	29
			Holl (ext)	23
			Ostende (ext)	28
			Wim (ext)	29
			Cha (int)	12
		edu_eu_north	Bodo	38
			Berg	38
gallo	gallo_atl	gallo_atl_iber	CAD	15
			COR	15
			CRE	15
			DKL	10
			Far	8
			FAR	14
			ORT	15
			Peniche	15
			VHO	15
			VIG	15
		gallo_atl_brit	Prim	36
	gallo_med	gallo_med_east	Croa	14
			Her	35
			MAK	6
		gallo_med_west	Can	3
			Th16	23
			TH16B	18
			Bizerte	12
tros	tros_am	tros_am	LochEti	11
			StLau-CBD	9
			StLau-TD	11
	tros_eu	tros_eu_baltic	Gdansk	12
	tros_pac	tros_pac_west	JapSea	16

Table S5: Linkage map – (i) genetic map produced from lab F2 crosses, (ii) markers on the same contig and (iii) left markers with unknown relation.

	locus	linkage group	position (cM)	unlinked set
(i)	063	1	0.00	•
	067	1	22.88	
	607	1	24.23	
	062	1	25.11	•
	210	1	28.33	
	055	1	28.77	
	034	1	31.99	
	115	1	32.44	
	064	2	0.00	
	026	2	1.36	
	047	2	1.36	
	160	2	1.36	•
	161	2	3.65	
	052	3	0.00	•
	007	3	9.70	
	610	3	27.41	
	015	4	0.00	
	155	4	2.29	•
	900	4	14.32	
	142	5	0.00	
	148	5	3.22	
	184	5	3.22	•
	061	6	0.00	
	080	6	10.00	•
	082	7	0.00	•
	802	7	10.00	
	144	8	0.00	
	145	8	10.00	•
	001	9	0.00	•
	022	10	0.00	•
	073	11	0.00	•
	094	12	0.00	•
	164	13	0.00	•
	180	14	0.00	•
	211	15	0.00	•
	617	16	0.00	•
(ii)	202	R_L02_mira_Contig1	2420 (bp)	•
	602	R_L02_mira_Contig1	3684 (bp)	
	604	R_L02_mira_Contig1	6232 (bp)	
(iii) All other markers not in the linkage map				•

General Note

p value	symbol
$p < 0.001$	***
$0.001 < p < 0.01$	**
$0.01 < p < 0.05$	*
$p > 0.05$	ns

When statistically significant, results are presented in bold.

Table S6: Fst values between reference groups.

Level	group 1	group 2	F_{ST}
L1	edu	gallo	**0.8127*****
	edu	tros	**0.7226*****
	gallo	tros	**0.8086*****
L2	gallo_med	gallo_atl	**0.3823*****
	edu_eu	edu_am	**0.4845*****
L3	gallo_med_west	gallo_med_east	**0.0586*****
	edu_eu_south_int	edu_eu_south_ext	0.0024 (ns)
	edu_eu_south_int	edu_eu_north	**0.2086*****
	edu_eu_south_ext	edu_eu_north	**0.3129*****
	gallo_atl_ext	gallo_atl_int	**0.1555*****

Table S7: Fst values and significance between ports.

	cher	havre	brest	stmalo
havre	**0.0235*****			
brest	**0.0046*****	**0.0245*****		
stmalo	0.0078 (ns)	**0.0390*****	0.0058 (ns)	
stnaz	0.0071 (ns)	**0.0304*****	**0.0067****	0.0043 (ns)

Table S8: Fst values and significance between Cherbourg years.

	cher03	cher15	cher16
cher15	**0.0066****		
cher16	**0.0097***	0.0009 (ns)	
cher17	0.0032 (ns)	-0.0017 (ns)	-0.0007 (ns)

Table S9: Fst values and significance between naturally and Norway admixed.

	ABD	HZSB	GAS	JER	LOF
HZSB	**0.0429*****				
GAS	**0.0736***	**0.1021***			
JER	**0.0974*****	**0.1439*****	0.0335 (ns)		
LOF	**0.0372*****	**0.0743*****	0.0047 (ns)	0.0414 (ns)	
MCH	0.0346 (ns)	0.0462 (ns)	0.1795 (ns)	**0.2330***	0.1163 (ns)

Table S10: Statistical tests of the differences in ancestry estimates between populations of implicating Atl. *M. galloprovincialis* and *M. edulis* (i.e. Norway admixed and naturally admixed). The Dwass-Steel-Crichtlow-Fligner test is used to compute post-hoc pairwise comparisons, the statistic (W) and corrected *p*-values for multiple tests are reported for each type of ancestry.

group1	group2	edu_eu_south		gallo_atl		gallo_med		edu_am	
		W	p.value	W	p.value	W	p.value	W	p.value
JER	ABD	-8.0014855	0.0000003	8.093009	0.0000003	-1.9301499	1.0000000	0.4648148	1.0000000
JER	MCH	-6.3869294	0.0000784	6.386518	0.0000392	-3.3998864	0.8073574	-1.2188032	1.0000000
JER	HZSB	-8.2758887	0.0000002	8.291331	0.0000002	-2.6247521	1.0000000	1.2462346	1.0000000
ABD	MCH	-5.7267576	0.0004261	5.920113	0.0001286	-2.5251363	1.0000000	-1.6665649	1.0000000
ABD	HZSB	-4.2334401	0.0152819	4.159408	0.0135876	-0.5622536	1.0000000	1.5813721	1.0000000
MCH	HZSB	1.5074295	1.0000000	-1.750562	0.7669834	2.3974014	1.0000000	2.9963772	0.1888831
GAS	LOF	-1.3324952	1.0000000	1.675714	0.7832573	-0.4038931	1.0000000	1.4839329	1.0000000
GAS	JER	5.8227527	0.0003818	-3.283418	0.0776347	0.9194070	1.0000000	-5.9576512	0.0001795
GAS	ABD	-1.2763083	1.0000000	6.491894	0.0000315	-0.3242880	1.0000000	-6.6249430	0.0000233
GAS	MCH	-4.5553253	0.0090960	6.585699	0.0000296	-2.0567182	1.0000000	-4.8952169	0.0033482
GAS	HZSB	-3.0020824	0.1682154	6.555299	0.0000296	-0.6873292	1.0000000	-7.1242722	0.0000059
LOF	JER	7.9519649	0.0000003	-5.949607	0.0001286	1.9547265	1.0000000	-8.4598018	0.0000000
LOF	ABD	0.7416394	1.0000000	6.047916	0.0001051	0.4258784	1.0000000	-10.4083896	0.0000000
LOF	MCH	-4.4370894	0.0106137	7.028091	0.0000090	-2.0570260	1.0000000	-6.6559353	0.0000233
LOF	HZSB	-2.5689731	0.3136611	7.008418	0.0000090	-0.0492258	1.0000000	-12.0687157	0.0000000

Table S11: Comparisons of ancestry estimates in dock mussel populations. See Table S10 for details.

group1	group2	edu_eu_south		gallo_atl		gallo_med		edu_am	
		W	p.value	W	p.value	W	p.value	W	p.value
brest	havre	10.7655111	0.0000000	-15.6453266	0.0000000	-3.341636	0.0714903	5.979967	0.0006899
brest	cher	0.5032010	1.0000000	-10.9995502	0.0000000	6.803901	0.0000110	2.996186	0.1429073
brest	stnaz	0.9072883	1.0000000	-5.6694271	0.0003583	3.482114	0.0674667	-1.141112	1.0000000
brest	stmalo	-2.9745824	0.1482498	-3.4396767	0.0627953	5.240644	0.0012351	-2.577176	0.2506348
havre	cher	-13.4998443	0.0000000	6.6257054	0.0000222	13.215188	0.0000000	-3.266336	0.1084834
havre	stnaz	-7.7970358	0.0000003	6.6026763	0.0000222	7.137681	0.0000044	-5.056126	0.0038002
havre	stmalo	-8.2353098	0.0000001	4.5053667	0.0070604	7.675113	0.0000008	-5.016890	0.0038002
cher	stnaz	0.8986060	1.0000000	2.4963584	0.2841525	-1.389627	0.9541097	-3.234122	0.1084834
cher	stmalo	-3.6887206	0.0511747	1.8065668	0.6554651	3.202155	0.0767377	-3.980020	0.0358256
stnaz	stmalo	-3.6202551	0.0511747	0.3570753	1.0000000	3.302863	0.0714903	-1.562515	0.8759137

Table S12: Comparisons of ancestry estimates in Cherbourg temporal sampling. See Table S10 for details.

group1	group2	edu_eu_south		gallo_atl		gallo_med		edu_am	
		W	p.value	W	p.value	W	p.value	W	p.value
cher17	cher16	-2.6860867	0.4230421	2.3094012	0.5026203	1.8889989	0.6677609	1.5834060	1
cher17	cher03	1.6852922	0.8582597	1.6552149	0.8892385	-2.6432869	0.3065542	0.3310927	1
cher17	cher15	0.4714781	1.0000000	-1.4395540	0.9074246	0.4614467	1.0000000	0.2006680	1
cher16	cher03	3.7937987	0.1074177	0.4877319	1.0000000	-4.2160701	0.0422489	-0.8576754	1
cher16	cher15	2.4189968	0.4273574	-3.1784465	0.3622642	-0.8510679	1.0000000	-0.7791183	1
cher03	cher15	-1.1499584	1.0000000	-2.4254679	0.5026203	2.6344868	0.3065542	-0.0209115	1

Table S13: Comparisons of ancestry estimates in Atl. *M. galloprovincialis* from Brittany. See Table S10 for details.

group1	group2	edu_eu_south		gallo_atl		gallo_med		edu_am	
		W	p.value	W	p.value	W	p.value	W	p.value
gallo_atl_brest_bay	gallo_atl_brest	-4.540429	0.0024335	4.074363	0.0072794	2.8645769	0.2354177	-1.909974	0.3243082
gallo_atl_brest_bay	gallo_atl_int	-12.819450	0.0000000	12.408811	0.0000000	-0.3787987	1.0000000	-8.974752	0.0000000
gallo_atl_brest	gallo_atl_int	-10.709842	0.0000000	10.484107	0.0000000	-2.4195419	0.2394843	-7.325735	0.0000006

Table S14: Estimation of the number of generations since admixture in ports.

port	average r	sd(r)	generations
brest	0.05873	0.00007	5.87
cher	0.07082	0.00006	7.08
cher03	0.12713	0.00061	12.71
cher15	0.07670	0.00031	7.67
cher16	0.06290	0.00008	6.29
cher17	0.06472	0.00013	6.47
havre	0.13902	0.00018	13.90
stmalo	0.08277	0.00066	8.28
stnaz	0.03472	0.00005	3.47

References

Alexander, D. H., & Lange, K. (2011). Enhancements to the ADMIXTURE algorithm for individual ancestry estimation. *BMC Bioinformatics*, *12*(1), 246. doi:10.1186/1471-2105-12-246

Analytics, R., & Weston, S. (2017). doMC: Foreach Parallel Adaptor for 'parallel' (Version R package version 1.3.5).

Anderson, E. C., & Thompson, E. A. (2002). A Model-Based Method for Identifying Species Hybrids Using Multilocus Genetic Data. *Genetics*, *160*(3), 1217–1229.

Broman, K. W., Wu, H., Sen, S., & Churchill, G. A. (2003). R/qtl: QTL mapping in experimental crosses. *Bioinformatics*, *19*, 889–890.

Coolen, J. W. P. (2017). *North Sea reefs: Benthic biodiversity of artificial and rocky reefs in the southern North Sea* (PhD thesis, Wageningen University, Wageningen).

Derryberry, E. P., Derryberry, G. E., Maley, J. M., & Brumfield, R. T. (2014). Hzar: Hybrid zone analysis using an R software package. *Molecular Ecology Resources*, *14*(3), 652–663. doi:10.1111/1755-0998.12209

Falush, D., Stephens, M., & Pritchard, J. K. (2003). Inference of Population Structure Using Multilocus Genotype Data: Linked Loci and Correlated Allele Frequencies. *Genetics*, *164*(4), 1567–1587.

Gompert, Z., & Buerkle, C. A. (2010). Introgress: A software package for mapping components of isolation in hybrids. *Molecular Ecology Resources*, *10*(2), 378–384. doi:10.1111/j.1755-0998.2009.02733.x

Goudet, J., & Jombart, T. [Thibaut]. (2015). Hierfstat: Estimation and Tests of Hierarchical F-Statistics.

Jombart, T. [T.], & Ahmed, I. (2011). Adegenet 1.3-1: New tools for the analysis of genome-wide SNP data. *Bioinformatics*, *27*(21), 3070–3071. doi:10.1093/bioinformatics/btr521

Jombart, T. [Thibaut]. (2008). Adegenet: A R package for the multivariate analysis of genetic markers. *Bioinformatics*, *24*, 1403–1405. doi:10.1093/bioinformatics/btn129

Kassambara, A. (2018). Ggpubr: 'ggplot2' Based Publication Ready Plots (Version R package version 0.2).

Kopelman, N. M., Mayzel, J., Jakobsson, M., Rosenberg, N. A., & Mayrose, I. (2015). Clumpak: A program for identifying clustering modes and packaging population structure inferences across K. *Molecular Ecology Resources*, *15*(5), 1179–1191. doi:10.1111/1755-0998.12387

Mathiesen, S. S., Thyrring, J., Hemmer-Hansen, J., Berge, J., Sukhotin, A., Leopold, P., ... Nielsen, E. E. (2016). Genetic diversity and connectivity within *Mytilus* spp. in the subarctic and Arctic. *Evolutionary Applications*, *10*, 39–55. doi:10.1111/eva.12415

Microsoft, & Weston, S. (2017). Foreach: Provides Foreach Looping Construct for R (Version R package version 1.4.4).

Paradis, E. (2010). Pegas: An R package for population genetics with an integrated–modular approach. *Bioinformatics*, *26*, 419–420.

Patil, I. (2019). Ggstatsplot: 'ggplot2' Based Plots with Statistical Details (Version R package version 0.0.8).

Poole, W., Gibbs, D. L., Shmulevich, I., Bernard, B., & Knijnenburg, T. A. (2016). Combining dependent *P*-values with an empirical adaptation of Brown's method. *Bioinformatics*, *32*(17), i430–i436. doi:10.1093/bioinformatics/btw438

Pritchard, J. K., Stephens, M., & Donnelly, P. (2000). Inference of population structure using multilocus genotype data. *Genetics*, *155*(2), 945–959. doi:10.1111/j.1471-8286.2007.01758.x

R Core Team. (2019). *R: A Language and Environment for Statistical Computing.* Vienna, Austria: R Foundation for Statistical Computing.

Revelle, W. (2018). Psych: Procedures for Psychological, Psychometric, and Personality Research (Version R package version 1.8.12). Evanston, Illinois.

Shin, J.-H., Blay, S., McNeney, B., & Graham, J. (2006). LDheatmap: An R Function for Graphical Display of Pairwise Linkage Disequilibria Between Single Nucleotide Polymorphisms. *J Stat Soft*, *16*, Code Snippet 3.

van Etten, J. (2017). R Package gdistance: Distances and Routes on Geographical Grids. *Journal of Statistical Software*, *76*(13). doi:10.18637/jss.v076.i13

Warnes, G., Gorjanc, w. c. f. G., Leisch, F., & Man, M. (2013). Genetics: Population Genetics (Version R package version 1.3.8.1).

Wickham, H. (2007). Reshaping Data with the reshape Package. *Journal of Statistical Software*, *21*(12), 1–20.

Wickham, H. (2017). Tidyverse: Easily Install and Load the 'Tidyverse' (Version R package version 1.2.1).
